# Fucosylated Chondroitin Sulfates from the Body Wall of the Sea Cucumber *Holothuria forskali*: CONFORMATION, SELECTIN BINDING, AND BIOLOGICAL ACTIVITY*[Fn FN2]

**DOI:** 10.1074/jbc.M114.572297

**Published:** 2014-08-21

**Authors:** Charalampos G. Panagos, Derek S. Thomson, Claire Moss, Adam D. Hughes, Maeve S. Kelly, Yan Liu, Wengang Chai, Radhakrishnan Venkatasamy, Domenico Spina, Clive P. Page, John Hogwood, Robert J. Woods, Barbara Mulloy, Charlie D. Bavington, Dušan Uhrín

**Affiliations:** From the ‡ EaStCHEM School of Chemistry, Joseph Black Building, The King's Buildings, University of Edinburgh, Edinburgh EH9 3JJ, United Kingdom,; § GlycoMar Ltd., European Centre for Marine Biotechnology, Dunstaffnage Marine Laboratory, Oban, Argyll PA37 1QA, United Kingdom,; the ¶ Scottish Association for Marine Science, Scottish Marine Institute, Oban, Argyll PA37 1QA, United Kingdom,; the ‖ Glycosciences Laboratory, Hammersmith Campus, Imperial College London, London W12 0NN, United Kingdom,; the ** Sackler Institute of Pulmonary Pharmacology, Institute of Pharmaceutical Science, King's College London, London SE1 9NH, United Kingdom,; the ‡‡ National Institute of Biological Standards and Controls, South Mimms, Potters Bar, Hertfordshire EN6 3QG, United Kingdom,; the §§ Complex Carbohydrate Research Center, the University of Georgia, Athens, Georgia 30602, and; the ¶¶ School of Chemistry, National University of Ireland Galway, University Road, Galway, Ireland

**Keywords:** Carbohydrate Structure, Carbohydrate-binding Protein, Glycosaminoglycan, Inflammation, Microarray, Molecular Dynamics, Nuclear Magnetic Resonance (NMR), GAG, Selectin

## Abstract

Fucosylated chondroitin sulfate (fCS) extracted from the sea cucumber *Holothuria forskali* is composed of the following repeating trisaccharide unit: →3)GalNAcβ4,6*S*(1→4) [Fucα*X*(1→3)]GlcAβ(1→, where *X* stands for different sulfation patterns of fucose (*X* = 3,4*S* (46%), 2,4*S* (39%), and 4*S* (15%)). As revealed by NMR and molecular dynamics simulations, the fCS repeating unit adopts a conformation similar to that of the Le^x^ blood group determinant, bringing several sulfate groups into close proximity and creating large negative patches distributed along the helical skeleton of the CS backbone. This may explain the high affinity of fCS oligosaccharides for L- and P-selectins as determined by microarray binding of fCS oligosaccharides prepared by Cu^2+^-catalyzed Fenton-type and photochemical depolymerization. No binding to E-selectin was observed. fCS poly- and oligosaccharides display low cytotoxicity *in vitro*, inhibit human neutrophil elastase activity, and inhibit the migration of neutrophils through an endothelial cell layer *in vitro*. Although the polysaccharide showed some anti-coagulant activity, small oligosaccharide fCS fragments had much reduced anticoagulant properties, with activity mainly via heparin cofactor II. The fCS polysaccharides showed prekallikrein activation comparable with dextran sulfate, whereas the fCS oligosaccharides caused almost no effect. The *H. forskali* fCS oligosaccharides were also tested in a mouse peritoneal inflammation model, where they caused a reduction in neutrophil infiltration. Overall, the data presented support the action of fCS as an inhibitor of selectin interactions, which play vital roles in inflammation and metastasis progression. Future studies of fCS-selectin interaction using fCS fragments or their mimetics may open new avenues for therapeutic intervention.

## Teaser

**Background:** An acidic polysaccharide, fCS, from the sea cucumber *Holothuria forskali* has a range of biological activities.

**Results:** The conformation of fCS was determined, and resulting oligosaccharides were shown to retain desirable biological properties.

**Conclusion:** The conformation of the fCS repeating unit underpins binding to L- and P-selectins.

**Significance:** Exploitation of the fCS-selectin interaction may open new avenues for therapeutic intervention using fCS fragments or their mimetics.

## Introduction

Sea cucumbers have been used as a traditional tonic food in many Asian countries for centuries, with the major edible parts being the body walls, which are predominantly composed of collagen and acidic polysaccharides. Structurally, the acidic body wall polysaccharides of sea cucumbers can be described as fucosylated chondroitin sulfates (fCS)^6^ (1–4) and sulfated fucans (5, 6). In addition to being a culinary delicacy, sea cucumbers have attracted considerable attention from researchers because of a range of biological activities in fCS that can be isolated from their tissues in high yields. fCS isolated from a variety of sea cucumbers were reported to possess anticoagulant (2, 7–14), antithrombotic (10, 15, 16), anti-inflammatory (17), anti-HIV (18–21), and metastasis blocking (22) properties.

The fCS polysaccharides isolated from sea cucumbers have a trisaccharide repeating unit with the following structure: →3)GalNAcβ(1→4) [Fucα(1→3)]GlcAβ(1→. The 2-O position of the glucuronic acid of the CS backbone repeating disaccharide is consistently free of substitutions, whereas the 6-O and 4-O positions of GalNAc can be either unsulfated or sulfated individually or simultaneously. The extent of GalNAc sulfation varies between species and/or different preparations; fCS isolated from *Ludwigothurea grisea* (2, 16, 23) and *Stichopus japonicus* (1, 24) contain 53 and 56% 6-O-sulfated species and 31 and 12% unsulfated GalNAc, respectively, whereas Yoshida *et al.* (4) isolated fCS from *S. japonicus* containing 100% GalNAcβ4,6*S* residues. Monofucosylation occurs invariably at the 3-O position of the glucuronic acids. The existence of extended fucose branches reported originally (23) has not been confirmed by subsequent studies. The fucose side chain can be either monosulfated (3-O or 4-O) or disulfated (2,4-O or 3,4-O), and the degree and pattern of sulfation varies between species (4, 8, 14, 16, 25).

The anticoagulant activity of fCS is linked with the presence of both the fucose branches and the sulfation of the main disaccharide repeating unit, as defucosylation or desulfation of the polysaccharide leads to the loss of its activity (26). Sulfated fucose on its own has no anticoagulant activity, and the positions of sulfates on both the CS backbone and the fucose side chain determine the anticoagulant potency of fCS (7, 8, 14, 25, 27). Generally, the depolymerized fragments of fCS have significantly decreased anticoagulant activity (28, 29).

In addition to interacting with anticoagulant proteins, fCSs also interact with the selectin family of cell adhesion molecules. fCS isolated from *L. grisea* is 4–8-fold more potent than heparin in inhibiting the interactions of P- and L-selectin with the sialyl Lewis x (sLe^x^) antigen. No inhibition of the interaction with E-selectin was observed (22). It was also suggested that attenuation of renal fibrosis in animal models by fCS is due to their binding to P-selectins (30).

As the first step toward rationalizing the various biological activities of fCS, we set out to determine the conformation of fCS and compare it with the known three-dimensional structures of linear glycosaminoglycans (GAGs) (27, 31, 32). We report here on the isolation of a GAG from the North Atlantic sea cucumber *Holothuria forskali* and its identification as an fCS by biochemical and biological analysis. In particular, we present a three-dimensional structure of the repeating unit of *H. forskali* fCS and show that (i) the conformation of the CS backbone of fCS is very similar to that of CS-A, (ii) fucose is stacked above the GalNAc residue of the preceding trisaccharide repeating unit in a manner seen between the fucose and galactose of the Le^x^ blood group trisaccharide, (iii) this arrangement is not affected by the sulfation pattern of fucose, and (iv) the resulting conformation creates a large concentration of negative charges on the surface of fCS. Using neoglycolipid-based oligosaccharide microarrays (33–35) we demonstrate strong binding of fCS oligosaccharides to L- and P-selectins. In addition, we report on the anticoagulant activity, prekallikrein activation, *in vitro* cell-based activity, and *in vivo* neutrophil recruitment of the *H. forskali* fCS and its fragments generated by two depolymerization methods.

## EXPERIMENTAL PROCEDURES

### 

#### 

##### Extraction and Purification of fCS

Individual samples of *H. forskali* were collected off the west coast of Scotland near Oban. The body wall of at least six individuals was separated from other components and used for GAG extraction. Extraction was carried out by a modification of previous methods (23). An equal volume of water was added to the tissue, and it was proteolytically digested overnight using alcalase (2.5L DX, Novozymes) at 1:100 (v/v), pH 8–9, and 60 °C. The resultant liquor was filtered and mixed with one-tenth volume of anion exchange resin (LEWATIT VPOC 1074/S6328A, 1:1 (v/v)) overnight. The resin was washed in water, and the bound material was eluted with 1 or 5 m NaCl and precipitated with 0.4 or 2 volumes of ethanol. The precipitates were air-dried, resuspended, dialyzed against water using 8-kDa MWCO tubing (BioDesign Inc.), and freeze-dried.

The four separated fractions were analyzed by HPLC-size exclusion chromatography using a Waters Alliance 2695 system (Waters (Manchester, UK) with refractive index and photodiode array detectors and a BioSep4000 column (300 × 7.8 mm, Phenomenex), calibrated with dextran standards (Fluka 12, 25, 50, 80, 270, and 670 kDa), and equilibrated in 50 mm Tris-HCl, pH 7, 1 mm EDTA, and 0.9% NaCl mobile phase. The uronic acid content was estimated by the carbazole reaction (36) and sulfation by a modified sulfate assay (37). These analyses indicated that only the 5 m NaCl 0.4-volume fraction was dominated by a highly sulfated component (>30% sulfate by molecular weight and ≥30% uronic acid content), which likely represented a GAG-like molecule (Table 1). Therefore, this fraction was further separated on a Sepacore preparative chromatography system (Buchi) using Q-Sepharose anion exchange resin (GE Healthcare) in an XK26/20 column. Gradient elution was carried out using 50 mm NaCl, 50 mm Tris-HCl, pH 7.5, 2 m NaCl, and 50 mm Tris-HCl, pH 7.5, running over 60 min with monitoring at A214 nm and conductivity. All peaks were collected, dialyzed (as above), and freeze-dried before repeating the above biochemical analysis. From this analysis a major peak eluting at 1.3–1.4 m NaCl was identified as likely containing a GAG-like molecule (>30% sulfate by molecular weight and ≥30% uronic acid content). Therefore it was selected for subsequent biochemical and biological analysis. Five repeat extraction batches confirmed the reproducibility of the described procedure (Table 1).

**TABLE 1:** **Summary of biochemical data for fractions collected during the extraction process**

Fractions	% Sulfate	Uronic acid*^a^*	Mass/kDa main peak*^b^*	% Area of main peak
	*S.D.*	*mg/ml*	*S.D.*	*S.D.*
1 m 0.4vol	14 (6.2, *n* = 4)	0.71 (*n* = 2)	<12 (*n* = 3)	80.4 (25.2, *n* = 3)
1 m 2 vol	LOQ*^c^* (*n* = 4)	0.45 (*n* = 2)	<12 (*n* = 4)	100 (0, *n* = 4)
5 m 0.4 vol	33 (2, *n* = 3)	0.54 (*n* = 2)	140 (11.5, *n* = 3)	93.9 (7.5, *n* = 3)
5 m 2 vol	18.7 (3.5, *n* = 4)	0.67 (*n* = 2)	<12 (*n* = 3)	100 (0, *n* = 3)
5 m 0.4 vol QS peak (1.4 m NaCl)*^d^*	33.4 (3, *n* = 5)	0.56 (0.05, *n* = 5)	137.9 (8.8, *n* = 5)	95 (10.7, *n* = 5)

*^a^* Carbazole method.

*^b^* Mass estimation based on HPLC-SEC with a dextran standard curve using refractive index detection.

*^c^* Lower limit of quantification (LOQ) of method is 3.8% sulfate.

*^d^* QS, Q-Sepharose.

##### Monosaccharide and Disaccharide Composition Analysis

Monosaccharide composition of the Q-Sepharose late-eluting fraction and of the Cu^2+^-catalyzed Fenton-generated depolymerized fragments (see below for preparation) was determined using a Shimadzu GC-2014 with flame ionization detection and ZB5-ms column. The samples (10 μl of a 10 mg/ml solution in water, dehydrated, washed with methanol, and dehydrated again) were subjected to methanolysis by the addition of 100 μl of 0.5 m methanolic-HCl (Supelco) at 85 °C for 4 h in heat-treated Reacti-Vials^TM^. This was followed by re-*N*-acetylation of free amines with the addition of 20 μl of neat acetic anhydride. Samples were dried, washed in methanol, and redried before the addition of 40 μl of neat trimethylsilane reagent (Supelco), mixing, and subsequent GC analysis. Mixed monosaccharide standards (5 nmol each) of arabinose, xylose, rhamnose, fucose, mannose, glucose, galactose, glucuronic acid, galacturonic acid, *N*-acetylgalactosamine, and *N*-acetylglucosamine (all Sigma) were run together with 5 nmol of scyllo-inositol (Sigma) as an internal control, which was also added to all samples to enable calculation of a standard ratio.

Disaccharide analysis was carried out on the Q-Sepharose fraction by digestion of a 1-mg of sample (100 μl of a 10 mg/ml solution in water) using either chondroitinase ABC lyase (12.5 milliunits/μl in 0.1 m ammonium acetate at 37 °C overnight) or heparinase II (both from Grampian Enzymes) (7 milliunits/μl in 5 mm Tris-HCl, pH 7, 50 mm NaCl, 0.1 mg/ml BSA at 25 °C overnight). The resulting digest was analyzed by HPLC-IEC using a Waters Alliance 2695 system with a ProPac PA1 column (4 × 250 mm, Dionex), running a water, pH 3.5/2 m NaCl, pH 3.5, gradient elution and photodiode array detection at 232 nm (38). Chondroitin (Di-0S, Di-4S, Di-6S, UA2S, Di-Se, Di-Sd, Di-Sb, or Di-TriS) or heparin (IVA, IVS, IIA, IIIA, IIS, IIIS, IA, or IS) disaccharide standards (Dextra Laboratories) were used, and chondroitin sulfate or heparin (Sigma) was run as a control, as well as no-enzyme controls for all samples.

##### NMR Spectroscopy

EDTA and trimethylsilylpropionate (TSP) were purchased from Goss Scientific Instruments Ltd. and Aldrich, respectively. The samples were dissolved in 540 μl of 99.9% D_2_O containing deuterated 10 mm NaH_2_PO_4_ + HNa_2_HPO_4_ (Sigma) buffer (pH 7.2) to which 20 μl of a stock solution of EDTA and TSP solution were added. The stock solution was prepared by dissolving 4 mg of EDTA and 9 mg of TSP in 200 μl of the phosphate buffer. The pH was adjusted to 7.2 by adding few drops of a concentrated solution of NaOH in D_2_O. All spectra were acquired at 50 °C on an 800 MHz Avance I (Bruker) NMR spectrometer equipped with a z-gradient triple resonance TCI cryoprobe. The spectra were referenced using ^1^H and ^13^C signals (0 ppm) of TSP.

^13^C one-dimensional NMR spectra were acquired using relaxation and acquisition times of 1.5 and 0.682 s; 14,080 scans were accumulated in 9 h per spectrum. FIDs (free induction decays) were zero-filled once, and a 2-Hz exponential line broadening was applied prior to Fourier transformation. Two-dimensional ^1^H-^13^C HSQC spectra were acquired using *T*_1_ and *T*_2_ acquisition times of 22 and 107 ms, respectively; 20 scans were acquired into each of 800 *F*_1_ complex data points, resulting in the total experimental times of 7 h/sample. The standard two-dimensional HSQC-TOCSY Bruker pulse sequence was modified by appending a ^1^H spin echo with an overall duration of 1/^1^*J*_CH_ (optimized for a ^1^*J*_CH_ = 150 Hz) after the TOCSY spin lock, and two two-dimensional ^1^H-^13^C HSQC-TOCSY spectra were acquired in an interleaved manner. The first spectrum with the 180° ^13^C pulse was applied simultaneously with the 180° ^1^H pulse of the final spin echo and the second one without. This resulted in a change of the sign of one-bond cross-peaks between the two spectra. The addition of the two two-dimensional matrixes prior to processing yielded a two-dimensional HSQC-TOCSY spectrum with substantially reduced one-bond cross-peaks. Such treatment facilitated the identification of weak TOCSY cross-peaks. Each two-dimensional HSQC-TOCSY spectrum was acquired using *T*_1_ and *T*_2_ acquisition times of 22 and 106 ms, respectively; 800 complex data points using 24 scans were collected in *F_1_* in each experiment; the total duration of one measurement was 18 h. Spin-lock was achieved via a DIPSI-2 mixing sequence applied for 25 ms. Two-dimensional ^1^H-^13^C, two-dimensional ^1^H-^13^C HSQC-NOESY spectra were acquired using *T*_1_ and *T*_2_ acquisition times of 21 and 106 ms, and 750 complex data points were collected in *F*_1_ using 192 scans. The total duration of the experiment was 66 h. The NOESY mixing time was set to 25 ms.

##### Molecular Dynamic Simulations

The initial three-dimensional structures of the fCS oligosaccharides were generated using the Carbohydrate Builder available on the GLYCAM Website. The templates generated were prepared for molecular dynamics (MD) simulation using the tLEaP module of AMBER 2014. All MD simulations were performed using the CUDA (39, 40) implementation of PMEMD in the AMBER 14 software suite (41). The carbohydrate parameters were taken from GLYCAM06j (42).

Prior to MD simulation, energy minimization was performed in implicit solvent (43) with Cartesian restraints (100 kcal/mol/Å^2^) on all residues. After such minimization, the system was solvated with explicit TIP3P water and reminimized in 5000 steps of steepest descent followed by 5000 steps of conjugate gradient using a nonbonding cutoff of 10.0 Å with no restraints.

The minimization steps were followed by a heating phase, during which the system was brought from 5 to 300 K over 50 ps. Finally, a production simulation of 50 ns was performed in the isothermal-isobaric ensemble, where the first 200 ps of the simulation were considered as the equilibration phase and excluded from subsequent analyses. Molecular graphics and analysis of the simulation results were performed with the UCSF Chimera package (44).

##### Depolymerization of the fCS Polysaccharide

The intact polymer was subjected to depolymerization using two different methods. The first was a Fenton-type free radical process using hydrogen peroxide and a copper catalyst that has been used previously to depolymerize fCS (45, 46). The native polymer (200 mg) was added to a reaction flask containing 100 ml of water. Copper acetate monohydrate was added to give a final concentration of 20 mm, and the reaction was heated to 60 °C and stabilized at pH 7 by the addition of sodium hydroxide. Hydrogen peroxide (2%) was pumped into the vessel at a rate of 22.5 ml/h to give a final peroxide:fCS ratio of 3.5 after a 90-min reaction time. These conditions generated oligosaccharide fragments of the desired target size. The reaction was maintained at pH 7 by the use of a pH control unit and sodium hydroxide. After depolymerization, excess copper was removed by Chelex (Sigma), and copper bound to the polymer was exchanged for sodium using Q-Sepharose (GE Healthcare) preparative chromatography in an XK26/20 column with 5 m NaCl as the eluent. Two size ranges of fragments were prepared using size exclusion preparative chromatography on a Superdex 30 (GE Healthcare) in an XK16/100 column with water as the mobile phase. These fragments were subjected to biochemical and biological analysis, as described above for the extraction process, to confirm the identity and evaluate any chain length-dependent biological effects. A list of samples and identifiers is shown in Table 2.

The fCS polysaccharide was also depolymerized using a novel photochemical-based method (47). fCS (300 mg) was added to water (30 ml) in a shallow crystallizing dish. 0.1% titanium(IV) oxide anatase powder was added, and a UV light (125-watt low pressure mercury lamp) was placed over the vessel as closely as possible to maximize UV absorbance by the titanium oxide. The reaction was left stirring for 3 days and was monitored by HPLC (Waters) using a Superdex peptide 10/300 GL (GE Healthcare) column. Fragments were separated using Bio-Gel P-10 resin (Bio-Rad Laboratories) for size exclusion chromatography using two XK26/100 columns and an XK26/20 guard column (GE healthcare). Their sizes were estimated using dermatan sulfate (DS) oligosaccharides (dp2–dp8, where dp is degree of polymerization) prepared by enzymatic depolymerization. The sample integrity was confirmed by NMR. The samples and identifiers are listed in Table 2.

##### Mass Spectrometry

Negative ion electrospray mass spectrometry of P-fCS-dp3 and P-fCS-dp4 was carried out on a Waters Synapt G2-S instrument. The oligosaccharide fractions were desalted on a gel filtration column Superdex peptide and eluted with ammonium acetate (0.05 mm). After removal of the volatile salt by repeated lyophilization, the sample was dissolved in water for analysis.

##### Preparation of Neoglycolipids (NGLs) and Detection of Selectin Binding by Microarray Analysis

The different fCS oligosaccharides used for selectin binding studies are shown in Table 2. Their concentration was determined by quantitation of hexuronic acid in a carbazole assay (36). Oligosaccharides from each fraction (∼50 nmol) were conjugated to amino-oxy-functionalized DHPE (Sigma) to generate NGLs by oxime ligation as described (33). Where indicated, groups of NGLs were subfractionated by semipreparative TLC (48). The following NGLs were used as controls: lacto-*N*-neotetraose, Galβ(1→4)GlcNAcβ(1→3)Galβ(1→4)Glc, 3′-sialyllacto-*N*-fucopentaose II (SA(3′)-LNFP-II), NeuAcα(2→3)Galβ(1→3)[Fucα(1→4)]GlcNAcβ(1→3)Galβ(1→4)Glc, 3′-sialyllacto-*N*-fucopentaose III (SA(3′)-LNFP-III), (NeuAcα(2→3)Galβ(1→4)[Fucα(1→3)]GlcNAcβ(1→3)Galβ(1→4)Glc (all from Dextra Laboratories), and CS-C hexasaccharide:ΔUA(1→3)GalNAc6*S*β(1→4)GlcAβ(1→3)GalNAc6*S*β(1→4)GlcAβ(1→3)GalNAc6*S* (49). These were prepared from the reducing oligosaccharides by reductive amination with DHPE (48). The NGLs of P-fCS-dp3, P-fCS-dp4 and P-fCS-dp6 were separated into upper and lower subfractions by preparative TLC using HPTLC plates with aluminum backing (5 μm, Merck) and the solvent system chloroform/methanol/water 60/35/8 (by volume). The NGL probes, 13 total, were robotically printed in duplicate on nitrocellulose-coated glass slides at two levels (2 and 5 fmol/spot) (34). The E-, P-, and L-selectins were analyzed as culture supernatants of IgM chimeras (50) at a 1/3 dilution, and their binding was detected with biotinylated anti-human IgM (Vector Laboratories) followed by Alexa Fluor-647-labeled streptavidin (Molecular Probes) at 1 μg/ml. The results, shown in Fig. 4, are representative of at least four analyses.

**TABLE 2:** **Identifiers of *H. forskali fCS* and fCS oligosaccharide samples used in this study** F, Fenton-type depolymerization; P, photochemical depolymerization; dp, degree of polymerization (an average polymer length).

Chemical and biological analysis	Selectin binding experiments
fCS (full-length polysaccharide)	F-fCS-dp10*^a^*
F-fCS-dp20*^b^*	F-fCS-dp6*^a^*
F-fCS-dp9*^b^*	P-fCS-dp10*^a^*
P-fCS-dp10*^a^*	P-fCS-dp6*^c^*
P-fCS-dp6*^a^*	P-fCS-dp4*^c^*
-	P-fCS-dp3*^c^*

*^a^* Fractionated on Bio-Gel P-10.

*^b^* Fractionated on Superdex 30.

*^c^* Fractionated on Bio-Gel P-10, separated into upper and lower fractions.

##### Anticoagulant Activity

Characterization of the anticoagulant activity of native fCS and the depolymerized fragments was performed using an automated coagulometer (Instrumentation Laboratories, Warrington, UK). Plasma-based methods included the European Pharmacopeia assay method for heparin sodium (01/2008:20705) and a second method, the activated partial thromboplastin time (APTT) assay. These two plasma methods differed, as the European Pharmacopeia method used sheep plasma (First Link Ltd.) and the second used human pooled plasma (National Blood Transfusion Service, UK). Antithrombin-dependent anti-factor Xa and anti-factor IIa assays were performed in accordance with the United States Pharmacopoeia monograph for heparin sodium (USP 34-NF 29), factor Xa from Diagnostic Reagents Ltd. (Oxfordshire, UK), and antithrombin and thrombin from the National Institute for Biological Standards and Control (NIBSC, UK). Heparin cofactor II (HCII, Enzyme Research Laboratories, Swansea, UK)-dependent inhibition of factor IIa was carried out by replacing antithrombin with HCII in the USP monograph method for anti-factor IIa activity. Potencies were assigned using parallel line analysis against either the Sixth International Standard for Unfractionated Heparin (07/328, NIBSC, UK) or the Second International Standard for Low Molecular Weight Heparin (LMWH).

##### Prekallikrein (PK) Activation

To further investigate the interaction of fCS with the contact activation pathway and inflammation, a PK activation assay was carried out. A dilution series of the samples (the native *H. forskali* fCS, F-fCS-dp20, and F-fCS-dp9 at 1.5–100 μg/ml) and a dextran sulfate (*Leuconostoc mesenteroides*, Sigma) positive control was set up, and each of the seven doses was mixed with normal pooled plasma (George King Bio-Medical Inc.) (51). After a 5-min activation period at 37 °C, a chromogenic substrate specific for plasma kallikrein was added (Chromogenix S2302), and color development, indicating substrate cleavage, was measured after 4 min (*A*_405–490 nm_). The change in absorbance was compared with a blank control related to the amount of kallikrein in the blood resulting from PK activation by test samples. Four runs were carried out, and a concentration-response curve was generated for each sample.

##### In Vitro Cell-based Activity

The cytotoxicity of *H. forskali* fCS, F-fCS-dp20, and F-fCS-dp9 was examined by measuring their effects on the metabolic activity of a BHK cell line (hamster kidney fibroblast, ECACC 85011433) cultured in Glasgow minimum essential medium with tryptase (Sigma), glutamine (GE Healthcare), and fetal calf serum (FCS) (Sigma). Triplicate wells of cells and samples (0.1 mg/ml) were incubated overnight at 37 °C in a 96-well plate, and metabolic activity was measured using CellTiter-Glo luminescent reagent (Promega) and a Synergy II plate reader (BioTek). Fucoidan (0.1 mg/ml, Marinova) and doxorubicin (0.01 and 0.001 mg/ml, Sigma) were run as assay controls, and % viability was calculated based on comparison with an untreated control.

The effect of the isolated fCS and of F-fCS-dp20 and F-fCS-dp9 oligosaccharides on neutrophil elastase activity was measured by incubation with freshly isolated human neutrophils. Test samples and a fucoidan control (0.1 mg/ml) were mixed with freshly isolated neutrophils, 5 μg/ml cytochalasin B (Sigma), and 10 ng/ml TNFα (Merck) and incubated for 30 min at 37 °C. 100 ng/ml fMLP (Sigma) was then added, and cells were further incubated for 45 min. Cells were removed by centrifugation and the supernatants mixed with 0.05 mg/ml elastase substrate (Merck) in triplicate wells of a 96-well microplate. A kinetic read was carried out on a Powerwave HT plate reader (BioTek) with measurements taken at 405 nm every 5 min for 1 h and *V*_max_ calculated for each sample. Elastase activity, given as %, was calculated by comparison with an untreated control.

The effect of the isolated fCS on the migration of neutrophils *in vitro* was measured using a chemotaxis assay. Human umbilical vein endothelial cells (PromoCell) were grown to 80% confluency on a 3-μm-pore Transwell insert in a 24-well tissue culture plate (Greiner), using PromoCell growth medium. Human umbilical vein endothelial cells were prestimulated with 0.01 μg/ml IL1β (Sigma) and 0.01 μg/ml TNFα (Merck) for 6 h. Freshly isolated human neutrophils stained with 2.5 μg/ml calcein (Sigma) and test compounds (1–100 μg/ml) were added above the membrane, and 100 ng/ml IL-8 (Sigma) was added below (in duplicate wells). After a 50-min incubation the Transwells were removed, and the number of calcein-labeled cells in the lower compartment were measured using a Synergy 2 plate reader (BioTek) at 485/528 nm. The % migration was calculated by comparison with an IL-8 only control.

##### Peritoneal Inflammation Model

To investigate the effect of *H. forskali* fCS on neutrophil recruitment to the peritoneal cavity of mice, a protocol was used based on Moffat *et al.* (52). Male BALBc mice (6–8 weeks, 20–22 g) were randomized to receive an intravenous injection of either saline control or test oligosaccharide (F-fCS-dp20 at 75 and 7.5 mg/kg and F-fCS-dp9 at 52 and 5.2 mg/kg) (5 animals/test group). 15 min after the introduction of vehicle or test saccharide, zymosan A (Sigma) was injected intraperitoneally to give a total dose of 1 mg/mouse. After 4 h mice were sacrificed, and 3 ml of saline was injected into the peritoneal cavity. The cavity was massaged, and 2 ml of lavage fluid was collected and stored on ice. The total and differential cells were enumerated to determine the effects of oligosaccharides on cell infiltration at the two doses tested.

## RESULTS

### 

#### 

##### fCS from H. forskali

**FIGURE 1.**
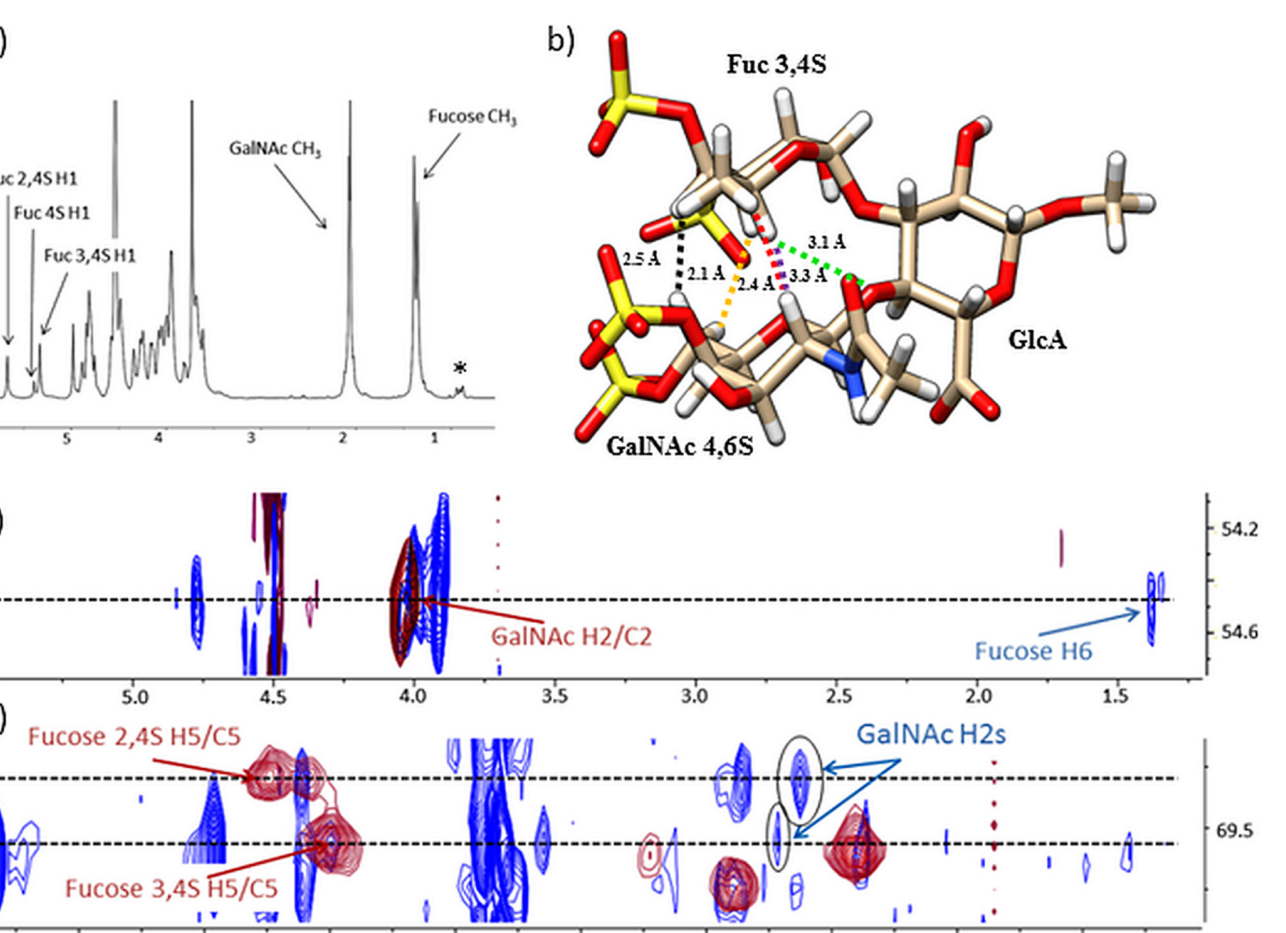
*a*, 800 MHz ^1^H NMR spectrum of *H. forskali* fCS. *b*, conformation of fCS **I**, highlighting the GalNAc/fucose protons showing inter-residue NOEs (H-2/H-5 (*purple*), H-6/H-3 (*yellow*), H-6/H-4 (*black*), and H-2/H-6 (*red*)), as well as the distance (*green*) between fucose H-5 and the oxygen involved in the GalNAcβ4,6*S*(1→4)GlcAβ glycosidic bond in both fCS **I** and fCS **II**. *c* and *d*, expansions of an overlay of the two-dimensional ^1^H-^13^C HSQC (*red*) and the two-dimensional ^1^H-^13^C HSQC-NOESY (*blue*) spectra. The inter-residue NOE cross-peaks are *circled*. The methyl resonances of proteins are marked by an *asterisk*.

The extraction of GAGs from the *H. forskali* body wall resulted in the isolation of a ∼95% pure peak by anion exchange chromatography (5 m 0.4-volume Q-Sepharose peak, Table 1). Biochemical analysis of this peak estimated it to be a highly sulfated molecule between 120 and 140 kDa (by HPLC-size exclusion chromatography using dextran standards). This is almost certainly an overestimation of molecular weight because of the difference in structure between standards and sample, but it provides a means of assessing batch-to-batch reproducibility at the extraction stage. A small amount of protein co-eluted with the fCS as indicated by the presence of small CH_3_ signals around ∼1.1 ppm in the ^1^H NMR spectrum (Fig. 1*a*). This impurity was not present in the depolymerized material.

Monosaccharide analysis of two preparations of this material suggested a composition of ∼7% rhamnose, 50% fucose, 2% galactose, 9% glucuronic acid, 30% *N*-acetylgalactosamine, and 2% galacturonic acid, strongly indicative of a fCS-like molecule. The low glucuronic acid content was likely due to the well documented limitations of acidic hydrolysis of GAGs (53), which can result in underestimation of the uronic acid content because of its resistance to hydrolysis or potential destruction when liberated. Equally other nonresistant components present in low amounts were probably overestimated (2% galactose, galacturonic acid, and 7% rhamnose) by this method, as they are not detectable by NMR. Nevertheless, the monosaccharide analysis provided strong evidence for a fucosylated GAG. No disaccharides were generated after chondroitinase ABC lyase or heparinase II enzymatic digestion of this peak, indicating that the product was resistant to digestion by these enzymes.

##### Primary Structure of the fCS Determined by NMR

Structural heterogeneity of the fCS isolated from *H. forskali* became evident from the inspection of its one-dimensional ^1^H spectrum (Fig. 1*a*). This spectrum contained three signals assignable to the anomeric protons of fucose, indicating different sulfation patterns of this residue. Nevertheless, it was possible to determine the primary structure of this polysaccharide through self-consistent analysis of two-dimensional ^1^H-^13^C HSQC, two-dimensional ^1^H-^13^C HSQC-TOCSY, and two-dimensional ^1^H-^13^C HSQC-NOESY 800-MHz spectra as →3)GalNAcβ4,6*S*(1→4)[Fucα*X*(1→3)]GlcAβ (1→. The *X* signifies the different sulfation patterns of fucose. On the other hand, only the 4,6-disulfation was found for the GalNAc residues. The three fucose sulfation patterns referred to as fCS **I–III**, are Fuc3,4*S* (**I**, 46%), Fuc2,4*S* (**II**, 39%), and Fuc4*S* (**III**, 15%) and represent the only heterogeneity of this fCS. The obtained ^1^H and ^13^C chemical shifts for all three forms are summarized in Table 3. Except for a constant difference of 2–2.5 ppm in the ^13^C spectrum caused by different referencing, these were remarkably consistent with those published previously by Yoshida *et al*. (4), who identified an fCS in the *S japonicus* sea cucumber, albeit with different ratios for sulfation patterns **I**–**III**. There is also a broad agreement of NMR data on fCS with a more recent work by Chen *et al.* (7).

**TABLE 3:**
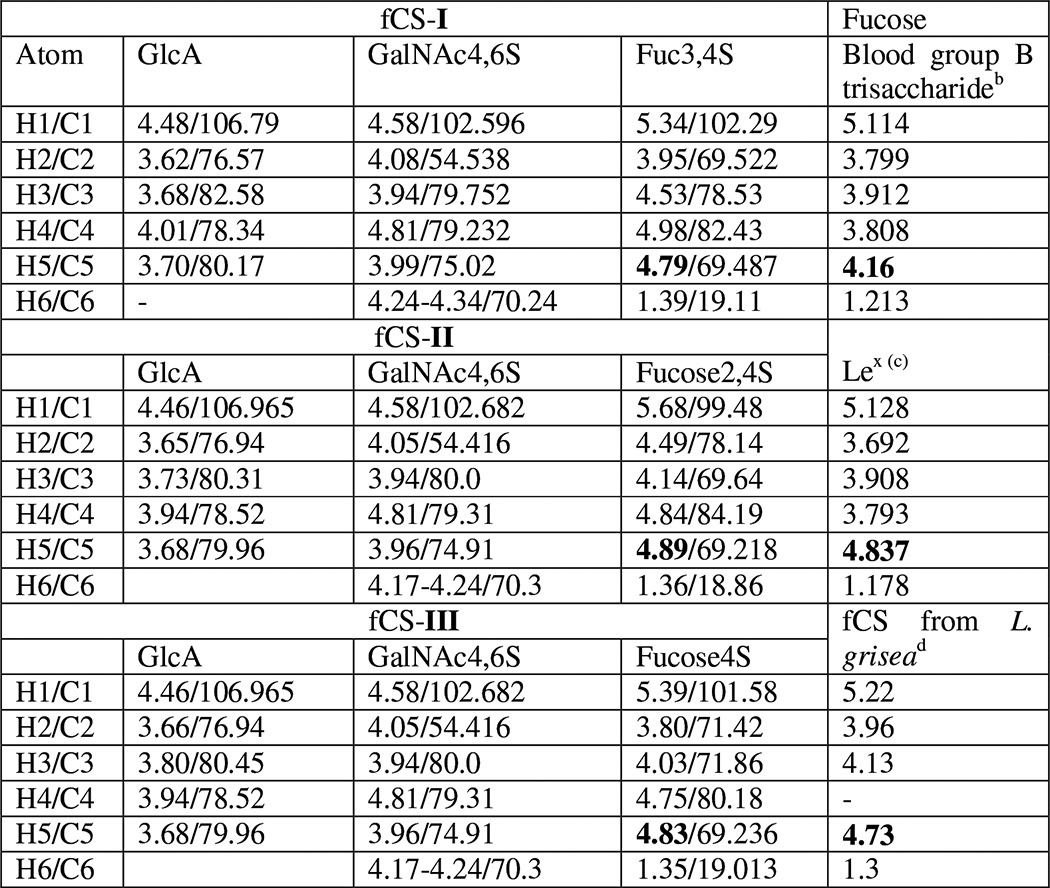
**^1^H and ^13^C chemical shifts (ppm) of fCS from *H. forskali* fCS and ^1^H chemical shifts of fucose-containing trisaccharides*^a^***

*^a^* The chemical shift of H5 of fucose is highlighted in bold; see “Results” for discussion.

*^b^* See Ref. 68.

*^c^* See Ref. 84.

*^d^* See Ref. 2.

##### Conformation of the Repeating fCS Trisaccharide Is Very Similar to That of the Le^x^ Trisaccharide

During the analysis of a two-dimensional ^1^H-^13^C HSQC-NOESY spectrum of *H. forskali* fCS, unusual cross-peaks were noticed between the protons of GalNAc and the fucose rings of fCS **I** (Fig. 1). The short interproton distances that gave rise to these cross-peaks indicates that the fucose residue is stacked on top of the GalNAc, as shown in Fig. 1*b*. This conformation is reminiscent of the conformation of the Le^x^ blood group determinant as determined by x-ray crystallography (54) and NMR spectroscopy (55). The Le^x^ trisaccharide, Galβ(1→4)[Fucα(1→3)]GlcNAcβ, is not dissimilar from that of the repeating unit of the fCS, →3)GalNAcβ4,6*S*(1→4)[Fucα*X*(1→3)]GlcAβ(1→. The differences in the primary structure between Le^x^ and fCS trisaccharides and the lack of sulfates in Le^x^ indicate that this rigid conformation is a consequence of similar carbohydrate scaffolds present in both species, *i.e.* similar monosaccharides composition, identical glycosidic linkages, and anomeric configurations, and is not disrupted by the sulfates of fCS.

##### The Ring Stacking Is Independent of the Sulfation Pattern

The same pattern of NOE peaks was also observed for fCS **II**, implying that the change of fucose sulfation from 3,4*S* to 2,4*S* did not affect the conformation of the trisaccharide fragment. The population of fCS **III** was too low to allow observation of NOE peaks in the ^13^C-edited NOESY experiment. Nevertheless, a credible indirect support for the existence of this conformation irrespective of the nature of the sulfation pattern came from the comparison of ^1^H chemical shifts of fucose (Table 3). The chemical shift of H-5 was particularly informative. In blood group B trisaccharide, in which fucose is linked to O-2 of galactose, the H-5 resonates at 4.16 ppm, whereas in the Le^x^ (the O-3-linked fucose) it is found at 4.84 ppm. The latter value was practically identical to the H-5 chemical shifts (4.79–4.89 ppm) in fCS **I–III**. The large deshielding of this proton is caused by stacking of the fucose residue on top of the preceding monosaccharide. The chemical shift of 4.73 ppm reported for the hydrolysis resistant part of *L. grisea* fCS (2) is also elevated. As this fCS contains primarily GalNAc6*S* and very little of the GalNAc4,6*S* species, we concluded that the stacking of fucose and GalNAc residues does not depend on the level of the GalNAc sulfation either.

It is interesting to note that a large chemical shift for fucose H-5 was also observed in the Le^a^ trisaccharide (56) in which fucose is (1→4)-linked, whereas Galβ is (1→3)-linked. Thus, despite swapping of the two linkages between Le^x^ and Le^a^, Fucα and Galβ are in juxtaposition in both trisaccharides (57, 58).

##### Molecular Dynamic Simulations of fCS I and II Oligosaccharides

The experimentally determined stacking of Fucα and GalNAcβ was also shown by MD using the GLYCAM06j force field with AMBER14. Such orientation was present 95% of the time for the MD simulation of fCS dodecasaccharides, fCS-dp12 (supplemental Figs. 1S and 2S and Table 1S). The distances between protons showing inter-residue NOEs were consistent with NMR data. The dihedral angles of Fucα(1→3)GlcAβ linkages were within 10° of those seen in an x-ray structure of Le^x^ (54) or a NMR solution structure of Le^x^ (59). The dihedral angles of GlcAβ(1→3)GalNAcβ and GalNAcβ (1→4)GlcAβ linkages were within 20° of the NMR solution structure of a CS pentasaccharide (60), desulfated CS hexasaccharide (61), or a CS tetrasaccharide in complex with chondroitinase B. The geometry of a GalNAcβ (1→4)GlcAβ linkage was markedly different from that of the x-ray structures of a CS fiber, which, however, is different from the values predicted by the exo-anomeric effect (62). This is likely caused by the presence of Ca^2+^ and/or crystal packing (63) of the x-ray structure. An inspection of the dihedral angles associated with the sulfate groups (supplemental Fig. 3S) showed that, with the exception of GalNAc C-6 sulfate, the orientation of the sulfate groups is relatively restrained, forming a well defined cluster of negative charge.

**FIGURE 2.**
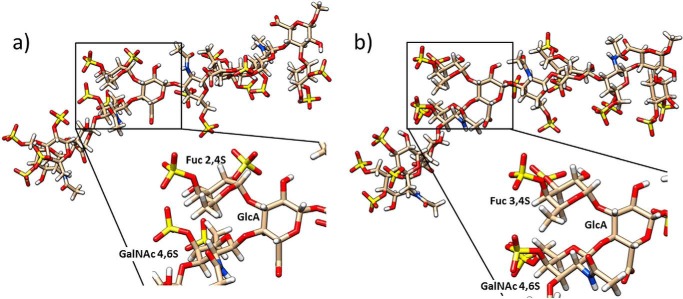
**Closest to average structures generated by MD simulations of 2,4 Fuc- (*a*) and 3,4 Fuc-sulfated fCS (*b*).** The *insets* show an expansion of the respective trisaccharide repeating units.

In summary, MD and NMR analysis revealed that fCS maintains the conformation of CS-A with fucose branches arranged in a Le^x^ manner (Fig. 2), and a high level of sulfation does not alter this conformation. As a consequence, the four sulfates of the repeating fCS trisaccharide are brought to close proximity, forming a large negative patch. This is the case for both sulfation patterns of fucose (fCS **I** and **II**).

The similarities in the conformation of fCS and that of Le^x^ (Le^a^) known to bind selectins prompted us to investigate the interaction of fCS with selectins. It is plausible that the rigid conformation common to these molecules and the subsequent spatial proximity of four sulfates are behind the binding of fCS to selectins (22). To study this interaction and the basic biological properties of fCS, oligosaccharides of fCS were prepared.

##### Depolymerization of the fCS

**FIGURE 3.**
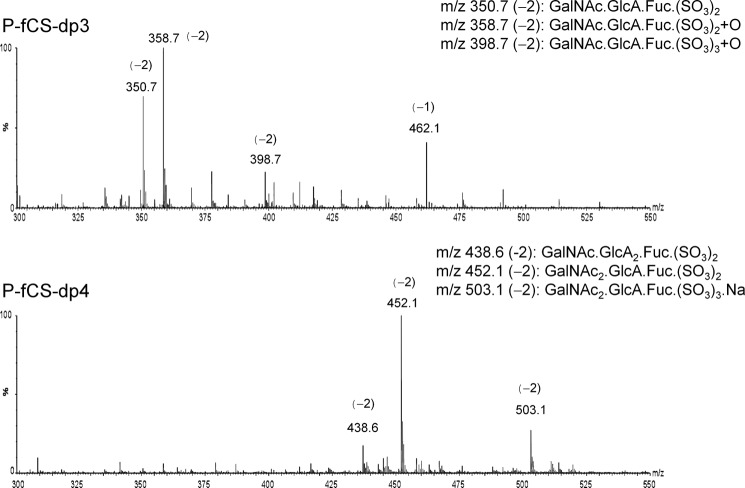
**Negative ion electrospray mass spectra of the tri- and tetrasaccharide fractions.**

Fenton-type Cu^2+^-catalyzed depolymerization using H_2_O_2_ radicals produced two size ranges of polymers; one averaged 6 kDa and the other 2.5 kDa, both estimated by HPLC-size exclusion chromatography using heparin standards. These sizes corresponded approximately to a 20-mer (F-fCS-dp20) and a nanosaccharide (F-fCS-dp9) containing ∼7 and 3 trisaccharide repeating units of fCS, respectively (Table 2). The photochemical depolymerization method also produced oligosaccharides of a similar size or smaller, compared with those generated by the Fenton-type method. Those used in this study were characterized, using DS oligosaccharide standards, as tri- to decasaccharides (P-fCS-dp3 to P-fCS-dp10) (Table 2). Using sulfated GAGs such as heparin or DS as molecular weight standards is potentially problematic when estimating the molecular weights of branched fCS oligosaccharides. We therefore determined the molecular weight of P-fCS-dp3 and P-fCS-dp4 fractions by MS and were able to confirm their correct classification (see below and Fig. 3). This is likely because the fucose branch is tightly packed against the main polysaccharide chain (Figs. 1 and 2).

The NMR analysis of P-fCS-dp3 and P-fCS-dp4 fractions (data not shown) indicated some structural heterogeneity, particularly associated with the fucose residue, which was likely because of the different sulfation patterns of this residue. The trisaccharide fraction contained only reducing end GlcA, implying the presence of GalNAcβ4,6*S* (1→4)[Fucα*X*(1→3)]GlcAβ trisaccharide. These data were corroborated by electrospray mass spectrometry with a trisaccharide composition of GalNAc, GlcA, and Fuc (molecular ion [M-2H]^2−^ at 350.7 (Fig. 3)).

Higher fractions are heterogeneous in sequence. In the case of the tetrasaccharide fraction P-fCS-dp4, an additional monosaccharide residue can be either a GlcA or GalNAc ([M-2H]^2−^ at 438.6 or 452.1), and the sequence can be either GlcAβ(1→3)GalNAcβ(1→4) [Fucα(1→3)]GlcAβ or GalNAcβ(1→4)[Fucα(1→3)]GlcAβ(1→3) GalNAcβ. The molecular ions detected by MS contained only 2–3 sulfate groups instead of the expected 4–6 groups. Inspection of the two-dimensional ^1^H-^13^C HSQC spectra of these fractions confirmed the presence of signals of sulfated residues, indicting some loss of sulfates during the MS experiments. In addition, both depolymerization processes lead to the opening of a proportion of the reducing GalNAc ring and formation of the galactosaminic acid by further oxidation, as evidenced also by the additional oxygen identified by MS (Fig. 3), similar to glucosaminic acid in heparin depolymerization reported previously (64, 65). Albeit heterogeneous, these oligosaccharides were considered suitable for initial selectin binding studies.

##### Binding of Selectins to fCS Oligosaccharides in Microarrays

**FIGURE 4.**
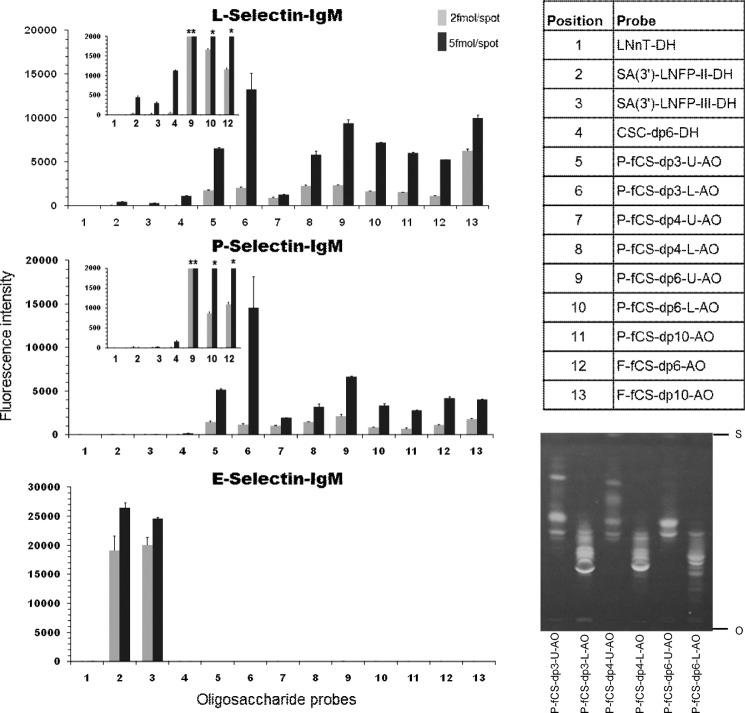
**Microarray analyses of the binding of human L-, P-, and E-selectins with NGLs derived from *H. forskali* fCS oligosaccharides.** The results shown are the means of the fluorescent intensities of duplicate spots at 2 and 5 fmol/spot with *error bars*. The *insets* in the L- and P-selectin panels are an expansion of the fluorescence signals of the control probes 1–4 in relation to the fCS hexasaccharide probes 9, 10, and 12.The *asterisks* indicate measured values that are off-scale. The details of fCS oligosaccharides are shown in Table 1, and sequences of the control NGLs are given under “Experimental Procedures.” High performance TLC of NGL fractions of P-fCS-dp3, -dp4, and -dp6 (*S*, solvent front; *O*, origin) is shown on the *bottom right*.

The fractions of *H. forskali* oligosaccharide fragments were converted into NGLs (33). During the purification process of NGLs it became apparent that each fraction contained multiple components, mainly because of a different degree of sulfation, in accord with the NMR and MS analyses above. The NGLs of P-fCS-dp3, P-fCS-dp4, and P-fCS-dp6 were separated into upper and lower subfractions by preparative TLC (Fig. 4). F-fCS-dp6 and F-fCS-dp10 fractions were also included in this study (see Table 2). Fig. 4 shows the results of the microarray analysis. In the present study, each oligosaccharide fraction was arrayed at two levels, 2 and 5 fmol/spot in duplicate. The binding strengths with the glycan probes immobilized at the two densities were dose-dependent. Several conclusions could be drawn from the data: (i) all fCS oligosaccharide fractions were bound by P- and L-selectins, whereas none was bound by E-selectin; (ii) signals elicited by all of the fCS fractions were stronger with L-selectin than with P-selectin; (iii) in both instances the binding intensities of the P-fCS-dp3-L fraction were comparable with those of larger oligosaccharide fractions. This suggests that the fCS trisaccharide unit, structurally similar to a Le^x^ trisaccharide, retains the affinity for P- and L-selectins. The lower apparent affinities of the tetrasaccharide and higher oligosaccharide fractions for selectins could be related to their heterogeneity not only in degree of sulfation but also in monosaccharide sequence (see above).A quantitative ranking of the binding strengths as a function of the chain length is therefore not possible at this point; (iv) fCS oligosaccharides were bound much more strongly by P- and L-selectins than SA(3′)-LNFP-II and SA(3′)-LNFP-III, which were included as positive controls, and the CS-C hexasaccharide, which lacks fucose. In a previous assay (data not shown) using P- and L-selectins a heparin 14-mer showed binding signals ∼2 times higher than SA(3′)-LNFP-II and SA(3′)-LNFP-III. This is in line with the inhibition of binding assays reported previously (66), which showed that heparin is a stronger binder of selectins. Our data therefore suggest that fCS oligosaccharides include components that are bound by P- and L-selectins with higher affinities than heparin or sLe^x^ (sLe^a^), and the fCS trisaccharide fraction contains components with comparable, even superior, binding compared with the longer species.

Following the microarray and the structural work, we further characterized the biological properties of the fCS polysaccharide and its oligosaccharides by standard biophysical and biochemical methods *in vitro* and *in vivo*. These data aimed to complement similar existing information on fCS polysaccharides isolated from a variety of sea cucumber species.

##### Anticoagulant Activities of H. forskali fCS

The estimated specific anticoagulant activities (in IU/mg) for *H. forskali* fCS are listed in Table 4. Antithrombin-dependent activities against both thrombin and factor Xa are very low, with activity at less than 1 IU/mg, whereas HCII-mediated activity is much higher, at 120 IU/mg, in keeping with that previously reported for these types of molecule (67). Clotting-based assays give values of 131 IU/mg in sheep plasma and 68.9 IU/mg in human plasma. In comparison, the fCS oligosaccharides derived by Fenton-type Cu^2+^-catalyzed or photochemical depolymerization showed an overall reduction from the parent fCS polysaccharide activity in all assays. The photochemically depolymerized fragments retain a higher anticoagulant activity than the chemically depolymerized fragments; P-fCS-dp10 APTT is 72.5 IU/mg, and F-fCS-dp9 APTT is 23.7 IU/mg (Table 4). HCII activity is more heavily reduced than APTT-based activity when the fCS is depolymerized; for example F-fCS-dp20 has a 15-fold decrease in HCII-mediated activity and only a 1.5-fold decrease by APTT measurement. Heparin (a normal clinical grade unfractionated heparin (NIBSC code 0/7/330) has the same level of activity in all the anticoagulant assays used. Oversulfated chondroitin sulfate (OSCS), purified from contaminated heparin (NIBSC code SS104), has the same activity profile as fCS except in the antithrombin-dependent assays. Here the contaminating heparin present in this OSCS sample caused higher readings. In the APTT and HCII assays the samples gave concentration-response curves parallel to that of the unfractionated heparin international standard, indicating that they behave in the same way as this standard when potency is estimated by these methods. However, when using the antithrombin methods this was not the case, and samples did not test parallel to the unfractionated or LMWH standards.

**TABLE 4:** **Estimated anticoagulant and antithrombotic potency values for *H. forskali* fCS** Values are in IU/1 mg of sample, with 95% confidence limits in parentheses. All were tested against the 6th International Standard for Unfractionated Heparin (07/328), except for the HCII assay, which was tested against the 2nd International Standard for LMWH (01/608). OSCS and heparin values are shown for comparison. EP, European Pharmacopeia; NIBSC, National Institute for Biological Standards and Control; USP, United States Pharmacopoeia; AT, antithrombin; LOD, limit of detection; F, Fenton-type depolymerization; P, photochemical depolymerization; dp, degree of polymerization (an average polymer length).

	Sheep plasma (EP) APTT	Human plasma (NIBSC) APTT	USP anti-Xa (AT-dependent)	USP anti-IIa (AT-dependent)	HCII (NIBSC)
Native fCS	131 (125–136)	68.9 (67.2–70.6)	0.40 (0.38–0.43)	0.56 (0.53–0.59)	120 (112–129)
F-fCS-dp20	89.5 (82.4–97.4)	24.4 (22.9–26.0)	0.46*^a^* (0.43–0.49)	<LOD	7.8 (6.6–9.5)
F-fCS-dp9	23.7 (21.9–25.6)	6.3 (6.1–6.6)	0.181*^a^* (0.176–0.185)	<LOD	0.65 (0.54–0.77)
P-fCS-dp10	72.5 (70.1–75.0)	51.4 (49.5–53.3)	1.10*^a^* (0.92–1.30)	<LOD	13.6*^b^* (12.3–15.0)
P-fCS-dp6	21.0 (15.7–28.1)	7.70*^c^* (7.43–7.98)	0.28*^a^* (0.26–0.29)	<LOD	0.96*^b^* (0.90–1.03)
OSCS	214 (207–222)	46.3 (45.2–47.3)	27.1 (25.6–28.9)	13.9 (13.2–14.7	201 (188–215)
Heparin	199 (189–209)	199 (193–204)	203 (187–219)	196 (176–218)	206 (197–216)

*^a^* Samples did not test parallel to the standard, indicating that they did not behave in the same manner.

*^b^* Samples tested well against the LMWH standard.

*^c^* The sample did not test well against the standard.

##### PK Activation

**FIGURE 5.**
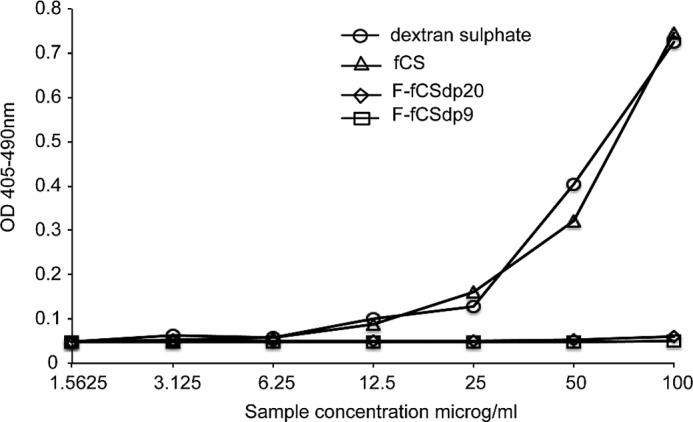
**Activation of PK by *H. forskali* fCS and fCS oligosaccharides.** The elevated OD represents the increasing presence of kallikrein in blood plasma, which is directly correlated with PK activation. Dextran sulfate, a known PK activator, was included for comparison. The native *H. forskali* fCS polysaccharide caused activation similar to that seen with dextran sulfate, but the oligosaccharides F-fCS-dp9 and F-fCS-dp20 did not activate PK at these concentrations.

*H. forskali* fCS and the oligosaccharides F-fCS-dp20 and F-fCS-dp9 were tested to assess their effects on the contact pathway by measuring PK activation. At doses above 12.5 μg/ml, the native fCS caused a large increase in plasma kallikrein, indicative of PK activation, which was comparable with that generated by a dextran sulfate positive control (Fig. 5). However, incubation of plasma with the fCS oligosaccharides caused almost no kallikrein increase at any of the seven concentrations tested, indicating that they did not cause PK activation. No standard or reference data were available, so the values presented are absolute.

##### In Vitro Cell-based activity of fCS and Its Fragments

The *H. forskali* fCS polysaccharide exhibited minimal cytotoxicity in a BHK cell viability assay at 100 μg/ml, with a cell viability of 87.5% (*n* = 5) as compared with an untreated control (Table 5). The depolymerized fragments also showed minimal effects on cell viability under these conditions with >93% (*n* = 6) cell viability (Table 5). The fCS was found to reduce human neutrophil elastase activity at the same concentration by ∼70% (Table 5). This could be either via a mechanism involving direct inhibition of the elastase enzyme or by inhibition of elastase release from the neutrophils. Human neutrophil elastase was also strongly inhibited by the depolymerized fragments with >84% inhibition (*n* = 6) (Table 5). F-fCS-dp20 oligosaccharides showed greater enzyme inhibition than the F-fCS-dp9 fraction, suggesting that chain lengths less than 6 kDa are less effective inhibitors. The native fCS was found to inhibit migration of neutrophils through an endothelial cell monolayer in a concentration-dependent manner, with ∼70% inhibition at 100 μg/ml, 50% at 10 μg/ml, and 10% at 1 μg/ml (Table 5).

**TABLE 5:**
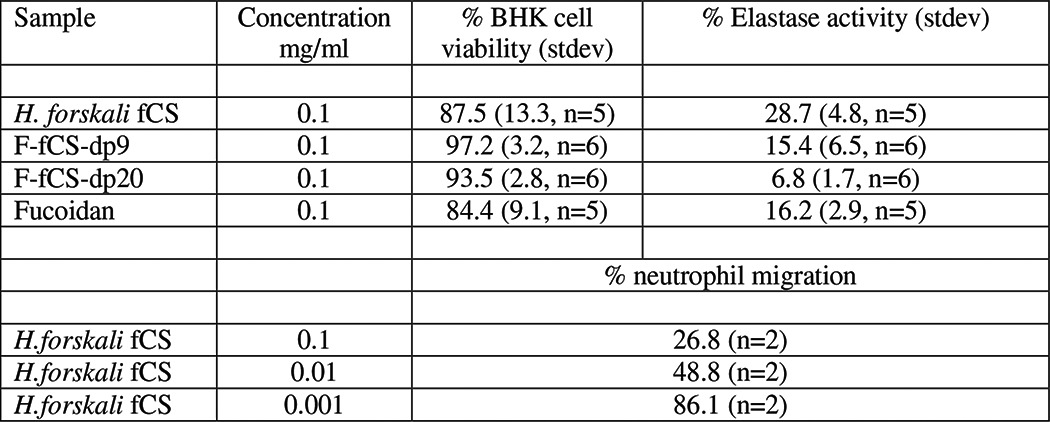
**Summary of *in vitro* biological activity of *H. forskali* fCS and fragments F-fCSdp9 and F-fCSdp20**

##### Peritoneal Inflammation

**FIGURE 6.**
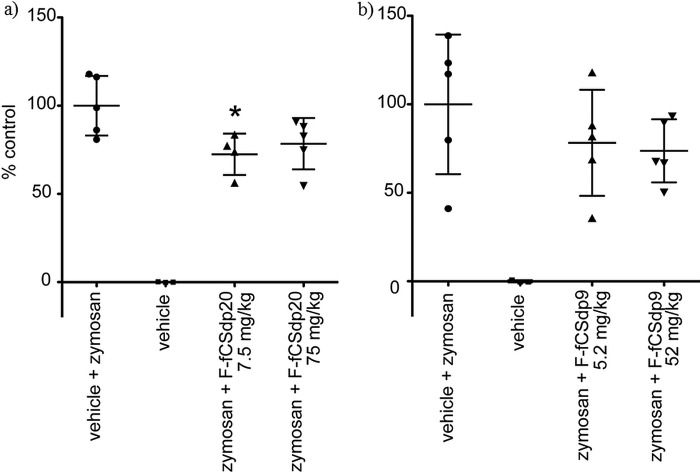
**Effects of *H. forskali* fCS oligosaccharides on zymosan-stimulated neutrophil recruitment to mouse peritoneal cavity (100% = vehicle + zymosan; 5 animals/test group).** The dexamethasone phosphate positive control tested under the same conditions exhibited 42% inhibition (±16%) with a *p* value of 0.059 (not shown). *a*, effect of F-fCS-dp20 on zymosan-stimulated neutrophil recruitment indicated an inhibitory effect at both doses but significance only in the lower dose (*, *p* < 0.05). *b*, effect of F-fCS-dp9 on zymosan-stimulated neutrophil recruitment indicated non-significant inhibitory effects at both doses.

The *H. forskali* fCS oligosaccharides were tested in a mouse peritoneal inflammation model. The stimulatory agent zymosan A caused a significant increase in neutrophil recruitment to the peritoneal cavity over controls, 74% (SD = 4%) and 79% (SD = 22%) in the two assay groups (Fig. 6). F-fCS-dp20 caused a significant inhibition (*p* = <0.05) of neutrophil recruitment to the peritoneal cavity at the lower dose of 7.5 mg/kg, amounting to ∼27% inhibition (Fig. 6*a*). The higher F-fCS-dp20 dose (75 mg/kg) and both doses of F-fCS-dp9 (5.2 and 52 mg/kg (Fig. 6*b*)) also caused a similar degree of inhibition of cell migration but did not achieve statistical significance. For comparison, a dexamethasone phosphate positive control tested under the same conditions resulted in a 42% (SD = 16%; not shown) inhibition of migration (although *p* = 0.059, thus just missing significance). The native fCS was also tested in this model under the same conditions, but it was found to have strong anticoagulant effects, and therefore the experiments were not pursued further.

## DISCUSSION

A polysaccharide isolated from the sea cucumber *H. forskali* was identified as an fCS and investigated in terms of biochemical and biological activity and by structural analysis using NMR spectroscopy. The biochemical characterization of the molecule by HPLC-size exclusion chromatography, sulfate assay, and monosaccharide and disaccharide analyses, all provided evidence for an fCS-like molecule, which was confirmed by NMR. The most surprising finding of the structural elucidation of fCS was the intermolecular NOE correlations observed in fCS, which revealed structural similarities of the branched trisaccharide repeating unit of fCS with the well characterized branched trisaccharide of the Le^x^ blood antigen determinant. We have demonstrated that the 3-O-GlcA-attached fucose of the fCSs trisaccharide repeating units is stacked above the GalNAc monosaccharide in a manner similar to that seen in the Le^x^, where the fucose is positioned above the Gal residue (54, 55). This is despite obvious differences in the primary structure of both carbohydrates.

Recently, an unconventional C-H…O bond between the H-5 of fucose of Le^x^ and O-5 of galactose was found by theoretical calculations to have a large stabilizing effect on such a conformation (68). The existence of this H-bond is supported by the elevated experimental chemical shifts of the H-5 of fucose in both of Le^x^ and fCS trisaccharide. Combined with van der Waals interactions, this could be the reason for stabilizing such conformation independently of the sulfation pattern of GalNAc or fucose and could potentially explain why both species bind to selectins. Nevertheless, the density of the negative charge is important for selectin binding, and highly sulfated linear GAGs have also been shown to bind to selectins. For example, affinity and kinetic analyses using surface plasmon resonance revealed that the oversulfated CS/DS chains containing GlcAβ(1→3)GalNAcβ4,6*S* disaccharide units bind to L- and P-selectins with high affinity (*K_D_* 21.1–293 nm). A CS tetrasaccharide containing two GalNAc4,6*S* residues also bound tightly to the selectins (69). In addition, the binding of sLe^x^ to P-selectin is much enhanced when this tetrasaccharide is part of a glycopeptide containing one or more tyrosine sulfate residues such as found in the PSGL-1 polypeptide (70, 71).

What are the underlying structural features of the fCS-selectin interaction? It is possible that the tight conformation of the fCS trisaccharide repeating unit, and the subsequent formation of a large negative patch, causes fCS to interact strongly with the two selectins. To determine whether this is the case, atomic resolution data of the fCS·L-/P-selectin complexes are required. Such data will also likely answer the question why structurally related E-selectin does not bind fCS or other sulfated GAGs such as heparin, whereas it does bind sLe^x/a^.

Because of the structural similarities between the fCS trisaccharide repeating unit and sLe^x^, as well as the existing literature on the binding of fCS polysaccharides with P- and L-selectins (22), small oligosaccharides were prepared from fCS and tested for selectin binding. As demonstrated by our NGL microarray analysis, there is a size-independent (up to dp10) tight binding of the small *H. forskali* fCS oligosaccharides to P- and L-selectins. This tight binding could explain the ability of fCS to inhibit selectin-Ig binding to immobilized PAA-sLe^x^ as observed previously (22). The blocking *in vitro* by fCS of the migration of human neutrophils through a vascular endothelial cell layer is supportive of the selectin binding studies, suggesting that this effect may have biological relevance, as observed for other GAGs (72, 73). This is also supported by our *in vivo* studies, which indicate that fCS oligosaccharide causes modest inhibition of zymosan-stimulated neutrophil migration into the mouse peritoneal cavity, likely to be through a selectin binding mechanism.

Even though the binding of the smaller fCS oligosaccharides (dp3–dp10) to selectins based on the microarray analysis appears to be size-independent, at least some of the biological activities of the native molecule are chain length-dependent. A reduction in polymer size appears to enhance elastase inhibition activity, although this effect is reversed once chain length drops below a critical size, possibly around 10 units. Conversely, anticoagulant activity is gradually lost as size decreases. An alternative interpretation for the decreased anticoagulation is a partial desulfation during the depolymerization process, but NMR analysis of the fragment generated by us, together with the data from the literature (45), indicate that this is not the case and the free radical depolymerization maintains the levels of sulfation of the native polymer. This is important, as it has been shown that the anticoagulant activity of fCS depends in part on the overall sulfation levels (28). NMR analysis of the photochemically depolymerized fCS oligosaccharides indicates a high degree of preservation of the original structural features. This provides further confidence that the biological activity observed is not due to any modifications resulting from the depolymerization process.

This apparent size and sulfation pattern-dependence of the anticoagulant activity of fCS is similar to that of other GAGs. The observation that with decreasing fCS polymer size, elastase inhibition activity is retained, but that anticoagulant activity is much reduced, could be of value in some therapeutic applications where anti-inflammatory effects are desirable but where anticoagulant activity would be unwanted. It could also reduce the adverse effects associated with the use of long, highly sulfated polysaccharide chains (74) as demonstrated in LMWH (75).

The anticoagulant action of the fCS polysaccharide has some similarities with OSCS, including sensitivity to the species origin (sheep or human) of the plasma used in the APTT assay (Table 4). The anticoagulant action of fCS is driven by potentiation of the HCII inhibition of thrombin, and there is no anticoagulant action mediated through antithrombin. This is similar to the response to OSCS, although the presence of heparin in this preparation gave a higher anti-thrombin-mediated activity. The depolymerized fragments of fCS have a marked reduction in HCII activity but retain, relatively speaking, a higher anticoagulant activity when measured by APTT. As this higher activity cannot be attributed to the potentiation of HCII inhibition (or antithrombin potentiation), there must be an alternative anticoagulant mechanism occurring. There is another observed pathway that is HCII-independent, where fCS acts by inhibiting factor X, through binding to the factor IXa-factor VIIIa complex (13), and factor XII (76).

The relative importance of the two anticoagulation pathways is debatable; Nagase *et al.* (13) suggest that the inhibition of factor X is of major importance for fCS hemostatic effects, whereas Fonseca *et al.* (9) report that fCS acts mainly through HCII but shows reduced anticoagulant activity when tested with antithrombin as the inhibitor and factor Xa as the target protease. This latter report is supported by the data presented in our current study. Experiments in rats have also shown differences in the plasma coagulation activity when the animals are injected intravenously with fCS or mammalian GAGs such as DS or LMWH (67).

Thus, it seems that the mechanisms of the anticoagulant activity of linear GAGs such as heparin, DS, or OSCS are different than that exhibited by branched sulfated polysaccharides such as fCS. This is likely to be a consequence of the differences between conformations of linear and branched GAGs, underpinned by different carbohydrate scaffolds and accentuated by specific distributions of the larger number of sulfate groups available. These factors may modulate the specificity and the strength of the interaction of GAGs with different proteins of the anticoagulation pathway, resulting in different biological properties of linear and branched sulfated GAGs.

It is interesting to note that in the case of HCII-dependent activity, the sulfation pattern of the fucose residue of fCS plays a major role in the activity of the polysaccharide, with Fuc2,4*S* exhibiting significantly higher anticoagulant activity than Fuc3,4*S* (8). On the other hand, in the case of HCII-independent binding, the sulfation pattern of the fucose of fCS does not affect the anticoagulant activity of the polysaccharide (7).

The structural similarities of the *H. forskali* fCS polysaccharide and the OSCS (the presence of the CS-E repeating disaccharides), together with similar anticoagulant activity profiles of these compounds, suggested that fCS may have the same pro-inflammatory effects as OSCS via the contact system/kinin-kallikrein pathway (77–79). Measurements of plasma kallikrein in an activation assay supported this theory by showing that the native polymer clearly causes PK activation at a level similar to dextran sulfate, although the shorter chain oligosaccharides do not. As the activation of PK is likely due to the high negative charge of the molecule, which allows it to form the required complex of PK, high molecular weight kininogen, and factor XII, it is presumed that the smaller oligosaccharide fragments do not carry a high enough net charge or size to enable complex formation, and thus no activation of PK takes place. This is in keeping with the other assays carried out in this study in that the native fCS has significant HCII activity, is toxic *in vivo*, and activates PK, whereas the fragments have no or minimal HCII activity, are non-toxic, have a mild anti-inflammatory effect *in vivo*, and do not activate PK.

The fCS polysaccharide and selected oligosaccharides were tested for their *in vitro* biological activities. Similar to the data observed for many GAGs, they displayed a low level of toxicity and inhibited the activity and/or the release of human neutrophil elastase (80–82). The fCS polysaccharide also inhibited the migration of human neutrophils through an endothelial cell layer *in vitro*, suggesting that it had properties similar to heparin-like GAGs (72) as well as other identified fCS (22).

In conclusion, the analysis of the fCS extracted from the sea cucumber *H. forskali* showed the presence of three different sulfation patterns of fucose, similar to those isolated from other sea cucumber species thus far. In addition, our NMR and MD studies showed that the repeating trisaccharide unit of fCS adopts a unique conformation in solution, similar to that of Le^x^ blood group trisaccharide determinant, whereas the backbone maintains the conformation of CS. As a direct consequence of this conformation, several sulfate groups of fCS are brought together forming a large negative patch. This, in combination with a rigid Le^x^-like conformation, may explain the high-affinity binding of fCS oligosaccharides to L- and P-selectins, which was established experimentally in microarray binding assays.

Although further targeted work with purified fCS oligosaccharides is required, the data presented in this study indicate that they are non-toxic *in vitro*, have low anticoagulant activity, do not activate prekallikrein, show some inhibition of the human neutrophil elastase activity, inhibit migration of neutrophils *in vitro* through an endothelial cell layer, and retain strong binding to L- and P-selectins. Atomic resolution data of the fCS·L-/P-selectin complexes are required to characterize this high-affinity binding in detail. Overall our data support of the action of fCS as an inhibitor of selectin interactions, which play vital roles in inflammation, the progression of metastasis, and potentially also in anti-HIV activities. It is also intriguing to note that the human dendritic lectin (DC-SIGN), involved in HIV entry, is able to bind Le^x^ (83), and therefore the anti-HIV properties of fCS could be linked to the competitive binding of the two molecules. Studies of fCS·selectin binding currently under way in our laboratory will thus hopefully open the way for a multitude of therapeutic interventions using fCS fragments or their mimetics.

## Supplementary Material

Supplemental Data

## Footnotes

6 The abbreviations used are:

fCS: fucosylated chondroitin sulfate
CS: chondroitin sulfate
GAG: glycosaminoglycan
TSP: trimethylsilylpropionate
MD: molecular dynamics
DS: dermatan sulfate
NGL: neoglycolipid
DHPE: 1,2-dihexadecyl-*sn*-glycero-3-phosphoethanolamine
APTT: activated partial thromboplastin time
LMWH: low molecular weight heparin
PK: prekallikrein
OSCS: oversulfated chondroitin sulfate.

## References

[1] Kariya Y. Watabe S. Hashimoto K. Yoshida K. (1990) Occurrence of chondroitin sulfate E in glycosaminoglycan isolated from the body wall of sea cucumber *Stichopus japonicus*. J. Biol. Chem. 265, 5081–5085 2108166

[2] Mourão P. A. Pereira M. S. Pavão M. S. Mulloy B. Tollefsen D. M. Mowinckel M. C. Abildgaard U. (1996) Structure and anticoagulant activity of a fucosylated chondroitin sulfate from echinoderm: sulfated fucose branches on the polysaccharide account for its high anticoagulant action. J. Biol. Chem. 271, 23973–23984 8798631 10.1074/jbc.271.39.23973

[3] Vieira R. P. Mourão P. A. (1988) Occurrence of a unique fucose-branched chondroitin sulfate in the body wall of a sea cucumber. J. Biol. Chem. 263, 18176–18183 3142869

[4] Yoshida K. Minami Y. Nemoto H. Numata K. Yamanaka E. (1992) Structure of DHG, a depolymerized glycosaminoglycan from sea cucumber *Stichopus japonicus*. Tetrahedron Lett. 33, 4959–4962

[5] Kariya Y. Mulloy B. Imai K. Tominaga A. Kaneko T. Asari A. Suzuki K. Masuda H. Kyogashima M. Ishii T. (2004) Isolation and partial characterization of fucan sulfates from the body wall of sea cucumber *Stichopus japonicus* and their ability to inhibit osteoclastogenesis. Carbohydr. Res. 339, 1339–1346 15113672 10.1016/j.carres.2004.02.025

[6] Ribeiro A. C. Vieira R. P. Mourão P. A. Mulloy B. (1994) A sulfated α-L-fucan from sea cucumber. Carbohydr. Res. 255, 225–240 8181009 10.1016/s0008-6215(00)90981-9

[7] Chen S. Li G. Wu N. Guo X. Liao N. Ye X. Liu D. Xue C. Chai W. (2013) Sulfation pattern of the fucose branch is important for the anticoagulant and antithrombotic activities of fucosylated chondroitin sulfates. Biochim. Biophys. Acta 1830, 3054–3066 23313164 10.1016/j.bbagen.2013.01.001

[8] Chen S. G. Xue C. H. Yin L. A. Tang Q. J. Yu G. L. Chai W. G. (2011) Comparison of structures and anticoagulant activities of fucosylated chondroitin sulfates from different sea cucumbers. Carbohydr. Polym. 83, 688–696

[9] Fonseca R. J. Oliveira S. N. Pomin V. H. Mecawi A. S. Araujo I. G. Mourão P. A. (2010) Effects of oversulfated and fucosylated chondroitin sulfates on coagulation: challenges for the study of anticoagulant polysaccharides. Thromb. Haemost. 103, 994–1004 20352164 10.1160/TH09-10-0734

[10] Fonseca R. J. Santos G. R. Mourão P. A. (2009) Effects of polysaccharides enriched in 2,4-disulfated fucose units on coagulation, thrombosis, and bleeding: practical and conceptual implications. Thromb. Haemost. 102, 829–836 19888516 10.1160/TH08-11-0773

[11] Glauser B. F. Pereira M. S. Monteiro R. Q. Mourão P. A. (2008) Serpin-independent anticoagulant activity of a fucosylated chondroitin sulfate. Thromb. Haemost. 100, 420–428 18766257

[12] Mourão P. A. Boisson-Vidal C. Tapon-Bretaudière J. Drouet B. Bros A. Fischer A. (2001) Inactivation of thrombin by a fucosylated chondroitin sulfate from echinoderm. Thromb. Res. 102, 167–176 11323028 10.1016/s0049-3848(01)00230-4

[13] Nagase H. Enjyoji K. Minamiguchi K. Kitazato K. T. Kitazato K. Saito H. Kato H. (1995) Depolymerized holothurian glycosaminoglycan with novel anticoagulant actions: antithrombin III- and heparin cofactor II-independent inhibition of factor X activation by factor IXa-factor VIIIa complex and heparin cofactor II-dependent inhibition of thrombin. Blood 85, 1527–1534 7888673

[14] Wu M. Huang R. Wen D. Gao N. He J. Li Z. Zhao J. (2012) Structure and effect of sulfated fucose branches on anticoagulant activity of the fucosylated chondroitin sulfate from sea cucumber *Thelenata ananas*. Carbohydr. Polym. 87, 862–868 10.1016/j.carbpol.2011.08.082 34663047

[15] Fonseca R. J. Mourão P. A. (2006) Fucosylated chondroitin sulfate as a new oral antithrombotic agent. Thromb. Haemost. 96, 822–829 17139379

[16] Mourão P. A. Guimarães B. Mulloy B. Thomas S. Gray E. (1998) Antithrombotic activity of a fucosylated chondroitin sulphate from echinoderm: sulphated fucose branches on the polysaccharide account for its antithrombotic action. Br. J. Haematol. 101, 647–652 9674735 10.1046/j.1365-2141.1998.00769.x

[17] Herencia F. Ubeda A. Ferrándiz M. L. Terencio M. C. Alcaraz M. J. García-Carrascosa M. Capaccioni R. Payá M. (1998) Anti-inflammatory activity in mice of extracts from Mediterranean marine invertebrates. Life Sci. 62, PL115–PL120 9496704 10.1016/s0024-3205(97)01188-0

[18] Beutler J. A. McKee T. C. Fuller R. W. Tischler M. Cardellina J. H. Snader K. M. McCloud T. G. Boyd M. R. (1993) Frequent occurrence of HIV-inhibitory sulphated polysaccharides in marine invertebrates. Antivir. Chem. Chemother. 4, 167–172

[19] Lian W. Wu M. Huang N. Gao N. Xiao C. Li Z. Zhang Z. Zheng Y. Peng W. Zhao J. (2013) Anti-HIV-1 activity and structure-activity relationship study of a fucosylated glycosaminoglycan from an echinoderm by targeting the conserved CD4-induced epitope. Biochim. Biophys. Acta 1830, 4681–4691 23769857 10.1016/j.bbagen.2013.06.003

[20] McClure M. O. Moore J. P. Blanc D. F. Scotting P. Cook G. M. Keynes R. J. Weber J. N. Davies D. Weiss R. A. (1992) Investigations into the mechanism by which sulfated polysaccharides inhibit HIV infection *in vitro*. AIDS Res. Hum. Retroviruses 8, 19–26 1346567 10.1089/aid.1992.8.19

[21] Huang N. Wu M. Y. Zheng C. B. Zhu L. Zhao J. H. Zheng Y. T. (2013) The depolymerized fucosylated chondroitin sulfate from sea cucumber potently inhibits HIV replication via interfering with virus entry. Carbohydr. Res. 380, 64–69 23962762 10.1016/j.carres.2013.07.010

[22] Borsig L. Wang L. Cavalcante M. C. Cardilo-Reis L. Ferreira P. L. Mourão P. A. Esko J. D. Pavão M. S. (2007) Selectin blocking activity of a fucosylated chondroitin sulfate glycosaminoglycan from sea cucumber: effect on tumor metastasis and neutrophil recruitment. J. Biol. Chem. 282, 14984–14991 17371880 10.1074/jbc.M610560200

[23] Vieira R. P. Mulloy B. Mourão P. A. (1991) Structure of a fucose-branched chondroitin sulfate from sea cucumber: evidence for the presence of 3-*O*-sulfo-β-d-glucuronosyl residues. J. Biol. Chem. 266, 13530–13536 1906878

[24] Kariya Y. Sakai T. Kaneko T. Suzuki K. Kyogashima M. (2002) Enhancement of t-PA-mediated plasminogen activation by partially defucosylated glycosaminoglycans from the sea cucumber *Stichopus japonicus*. J. Biochem. 132, 335–343 12153733 10.1093/oxfordjournals.jbchem.a003228

[25] Luo L. Wu M. Xu L. Lian W. Xiang J. Lu F. Gao N. Xiao C. Wang S. Zhao J. (2013) Comparison of physicochemical characteristics and anticoagulant activities of polysaccharides from three sea cucumbers. Mar. Drugs 11, 399–417 23385300 10.3390/md11020399 PMC3640388

[26] Mulloy B. Mourão P. A. Gray E. (2000) Structure/function studies of anticoagulant sulphated polysaccharides using NMR. J. Biotechnol. 77, 123–135 10674219 10.1016/s0168-1656(99)00211-4

[27] Mulloy B. Forster M. J. (2000) Conformation and dynamics of heparin and heparan sulfate. Glycobiology 10, 1147–1156 11087707 10.1093/glycob/10.11.1147

[28] Wu N. Ye X. Guo X. Liao N. Yin X. Hu Y. Sun Y. Liu D. Chen S. (2013) Depolymerization of fucosylated chondroitin sulfate from sea cucumber, *Pearsonothuria graeffei*, via Co-60 irradiation. Carbohydr. Polym. 93, 604–614 23499102 10.1016/j.carbpol.2012.12.044

[29] Wu M. Xua S. Zhao J. Kang H. Ding H. (2010) Physicochemical characteristics and anticoagulant activities of low molecular weight fractions by free-radical depolymerization of a fucosylated chondroitin sulphate from sea cucumber *Thelenota ananas*. Food Chem. 122, 716–723

[30] Melo-Filho N. M. Belmiro C. L. Gonçalves R. G. Takiya C. M. Leite M. Jr. Pavão M. S. Mourão P. A. (2010) Fucosylated chondroitin sulfate attenuates renal fibrosis in animals submitted to unilateral ureteral obstruction: a P-selectin-mediated event? Am. J. Physiol. Renal Physiol. 299, F1299–F1307 20861075 10.1152/ajprenal.00217.2010

[31] Jin L. Hricovíni M. Deakin J. A. Lyon M. Uhrín D. (2009) Residual dipolar coupling investigation of a heparin tetrasaccharide confirms the limited effect of flexibility of the iduronic acid on the molecular shape of heparin. Glycobiology 19, 1185–1196 19648354 10.1093/glycob/cwp105 PMC2757574

[32] Silipo A. Zhang Z. Cañada F. J. Molinaro A. Linhardt R. J. Jiménez-Barbero J. (2008) Conformational analysis of a dermatan sulfate-derived tetrasaccharide by NMR, molecular modeling, and residual dipolar couplings. Chembiochem 9, 240–252 18072186 10.1002/cbic.200700400 PMC4135520

[33] Liu Y. Feizi T. Campanero-Rhodes M. A. Childs R. A. Zhang Y. Mulloy B. Evans P. G. Osborn H. M. Otto D. Crocker P. R. Chai W. (2007) Neoglycolipid probes prepared via oxime ligation for microarray analysis of oligosaccharide-protein interactions. Chem. Biol. 14, 847–859 17656321 10.1016/j.chembiol.2007.06.009

[34] Liu Y. Childs R. A. Palma A. S. Campanero-Rhodes M. A. Stoll M. S. Chai W. Feizi T. (2012) Neoglycolipid-based oligosaccharide microarray system: preparation of NGLs and their noncovalent immobilization on nitrocellulose-coated glass slides for microarray analyses. Methods Mol. Biol. 808, 117–136 22057521 10.1007/978-1-61779-373-8_8

[35] Feizi T. (2013) Carbohydrate recognition in the immune system: contributions of neoglycolipid-based microarrays to carbohydrate ligand discovery. Ann. N.Y. Acad. Sci. 1292, 33–44 23834439 10.1111/nyas.12210 PMC4260124

[36] Cesaretti M. Luppi E. Maccari F. Volpi N. (2003) A 96-well assay for uronic acid carbazole reaction. Carbohydr. Polym. 54, 59–61

[37] Terho T. T. Hartiala K. (1971) Method for determination of the sulfate content of glycosaminoglycans. Anal. Biochem. 41, 471–476 4253906 10.1016/0003-2697(71)90167-9

[38] Turnbull J. E. (2001) Analytical and preparative strong anion-exchange HPLC of heparan sulfate and heparin saccharides. Methods Mol. Biol. 171, 141–147 11450224 10.1385/1-59259-209-0:141

[39] Götz A. W. Williamson M. J. Xu D. Poole D. Le Grand S. Walker R. C. (2012) Routine microsecond molecular dynamics simulations with AMBER on GPUs. 1. Generalized born. J. Chem. Theory Comput. 8, 1542–1555 22582031 10.1021/ct200909j PMC3348677

[40] Salomon-Ferrer R. Goetz A. W. Poole D. Le Grand S. Walker R. C. (2013) Routine microsecond molecular dynamics simulations with AMBER on GPUs. 2. Explicit solvent particle mesh Ewald. J. Chem. Theory Comput. 9, 3878–3888 10.1021/ct400314y 26592383

[41] Case D. A. Babin V. Berryman J. T. Betz R. M. Cai Q. Cerutti D. S. Cheatham T. E. 3rd Darden T. A. Duke R. E. Gohlke H. Goetz A. W. Gusarov S. Homeyer N. Janowski P. Kaus J. Kolossváry I. Kovalenko A. Lee T. S. LeGrand S. Luchko T. Luo R. Madej B. Merz K. M. Paesani F. Roe D. R. Roitberg A. Sagui C. Salomon-Ferrer R. Seabra G. Simmerling C. L. Smith W. Swails J. Walker R. C. Wang J. Wolf R. M. Wu X. Kollman P. A. (2014) AMBER 14, University of California, San Francisco

[42] Kirschner K. N. Yongye A. B. Tschampel S. M. González-Outeiriño J. Daniels C. R. Foley B. L. Woods R. J. (2008) GLYCAM06: A generalizable biomolecular force field: Carbohydrates. J. Comput. Chem. 29, 622–655 17849372 10.1002/jcc.20820 PMC4423547

[43] Case D. A. Cheatham T. E. 3rd Darden T. Gohlke H. Luo R. Merz K. M. Jr. Onufriev A. Simmerling C. Wang B. Woods R. J. (2005) The Amber biomolecular simulation programs. J. Comput. Chem. 26, 1668–1688 16200636 10.1002/jcc.20290 PMC1989667

[44] Pettersen E. F. Goddard T. D. Huang C. C. Couch G. S. Greenblatt D. M. Meng E. C. Ferrin T. E. (2004) UCSF chimera: a visualization system for exploratory research and analysis. J. Comput. Chem. 25, 1605–1612 15264254 10.1002/jcc.20084

[45] Wu M. Y. Xu S. M. Zhao J. H. Kang H. Ding H. (2010) Free-radical depolymerization of glycosaminoglycan from sea cucumber *Thelenota ananas* by hydrogen peroxide and copper ions. Carbohydr. Polym. 80, 1116–1124 10.1016/j.carres.2009.11.030 20117762

[46] Petit A. C. Noiret N. Sinquin C. Ratiskol J. Guezennec J. Colliec-Jouault S. (2006) Free-radical depolymerization with metallic catalysts of an exopolysaccharide produced by a bacterium isolated from a deep-sea hydrothermal vent polychaete annelid. Carbohydr. Polym. 64, 597–602

[47] Higashi K. Hosoyama S. Ohno A. Masuko S. Yang B. Sterner E. Wang Z. Linhardt R. J. Toida T. (2012) Photochemical preparation of a novel low molecular weight heparin. Carbohydr. Polym. 87, 1737–1743 22205826 10.1016/j.carbpol.2011.09.087 PMC3245882

[48] Chai W. Stoll M. S. Galustian C. Lawson A. M. Feizi T. (2003) Neoglycolipid technology: deciphering information content of glycome. Methods Enzymol. 362, 160–195 12968363 10.1016/S0076-6879(03)01012-7

[49] Chai W. Kogelberg H. Lawson A. M. (1996) Generation and structural characterization of a range of unmodified chondroitin sulfate oligosaccharide fragments. Anal. Biochem. 237, 88–102 8660542 10.1006/abio.1996.0205

[50] Galustian C. Childs R. A. Stoll M. Ishida H. Kiso M. Feizi T. (2002) Synergistic interactions of the two classes of ligand, sialyl-Lewis(*a*/*x*) fuco-oligosaccharides and short sulpho-motifs, with the P- and L-selectins: implications for therapeutic inhibitor designs. Immunology 105, 350–359 11918697 10.1046/j.1365-2567.2002.01369.x PMC1782666

[51] van der Graaf F. Keus F. J. Vlooswijk R. A. Bouma B. N. (1982) The contact activation mechanism in human plasma: activation induced by dextran sulfate. Blood 59, 1225–1233 6177360

[52] Moffatt J. D. Lever R. Page C. P. (2004) Effects of inhaled thrombin receptor agonists in mice. Br. J. Pharmacol. 143, 269–275 15302675 10.1038/sj.bjp.0705926 PMC1575335

[53] Manzi A. (1995) Acid hydrolysis for release of monosaccharides. Curr. Protoc. Mol. Biol. Supplement 32, **Chapter 17**, Unit 17.16.1–17.16.11 10.1002/0471142727.mb1716s32 18265147

[54] Pérez S. Mouhous-Riou N. Nifant'ev N. E. Tsvetkov Y. E. Bachet B. Imberty A. (1996) Crystal and molecular structure of a histo-blood group antigen involved in cell adhesion: The Lewis x trisaccharide. Glycobiology 6, 537–542 8877374 10.1093/glycob/6.5.537

[55] Poppe L. Brown G. S. Philo J. S. Nikrad P. V. Shah B. H. (1997) Conformation of sLe(x) tetrasaccharide, free in solution and bound to E-, P-, and L-selectin. J. Am. Chem. Soc. 119, 1727–1736

[56] Bechtel B. Wand A. J. Wroblewski K. Koprowski H. Thurin J. (1990) Conformational analysis of the tumor-associated carbohydrate antigen 19–9 and its Lea blood group antigen component as related to the specificity of monoclonal antibody CO19–9. J. Biol. Chem. 265, 2028–2037 2298737

[57] Kogelberg H. Frenkiel T. A. Homans S. W. Lubineau A. Feizi T. (1996) Conformational studies on the selectin and natural killer cell receptor ligands sulfo- and sialyl-lacto-*N*-fucopentaoses (SuLNFPII and SLNFPII) using NMR spectroscopy and molecular dynamics simulations: comparisons with the nonacidic parent molecule LNFPII. Biochemistry 35, 1954–1964 8639679 10.1021/bi9521598

[58] Lemieux R. U. Bock K. Delbaere L. T. Koto S. Rao V. S. (1980) The conformations of oligosaccharides related to the ABH and Lewis human blood group determinants. Can. J. Chem. 58, 631–653

[59] Azurmendi H. F. Martin-Pastor M. Bush C. A. (2002) Conformational studies of Lewis X and Lewis A trisaccharides using NMR residual dipolar couplings. Biopolymers 63, 89–98 11786997 10.1002/bip.10015

[60] Yu F. Wolff J. J. Amster I. J. Prestegard J. H. (2007) Conformational preferences of chondroitin sulfate oligomers using partially oriented NMR spectroscopy of C-13-labeled acetyl groups. J. Am. Chem. Soc. 129, 13288–13297 17924631 10.1021/ja075272h

[61] Sattelle B. M. Shakeri J. Roberts I. S. Almond A. (2010) A 3D-structural model of unsulfated chondroitin from high-field NMR: 4-sulfation has little effect on backbone conformation. Carbohydr. Res. 345, 291–302 20022001 10.1016/j.carres.2009.11.013 PMC3098369

[62] Michel G. Pojasek K. Li Y. Sulea T. Linhardt R. J. Raman R. Prabhakar V. Sasisekharan R. Cygler M. (2004) The structure of chondroitin B lyase complexed with glycosaminoglycan oligosaccharides unravels a calcium-dependent catalytic machinery. J. Biol. Chem. 279, 32882–32896 15155751 10.1074/jbc.M403421200 PMC4135467

[63] Cael J. J. Winter W. T. Arnott S. (1978) Calcium chondroitin 4-sulfate: molecular conformation and organization of polysaccharide chains in a proteoglycan. J. Mol. Biol. 125, 21–42 712856 10.1016/0022-2836(78)90252-8

[64] Beccati D. Roy S. Yu F. Gunay N. S. Capila I. Lech M. Linhardt R. J. Venkataraman G. (2010) Identification of a novel structure in heparin generated by potassium permanganate oxidation. Carbohydr. Polym. 82, 699–705 25147414 10.1016/j.carbpol.2010.05.038 PMC4138061

[65] Panagos C. Thomson D. Bavington C. D. Uhrin D. (2012) Structural characterisation of oligosaccharides obtained by Fenton-type radical depolymerisation of dermatan sulfate. Carbohydr. Polym. 87, 2086–2092

[66] Koenig A. Norgard-Sumnicht K. Linhardt R. Varki A. (1998) Differential interactions of heparin and heparan sulfate glycosaminoglycans with the selectins: implications for the use of unfractionated and low molecular weight heparins as therapeutic agents. J. Clin. Invest. 101, 877–889 9466983 10.1172/JCI1509 PMC508636

[67] Pacheco R. G. Vicente C. P. Zancan P. Mourão P. A. (2000) Different antithrombotic mechanisms among glycosaminoglycans revealed with a new fucosylated chondroitin sulfate from an echinoderm. Blood Coagul. Fibrinolysis 11, 563–573 10997797 10.1097/00001721-200009000-00009

[68] Zierke M. Smieško M. Rabbani S. Aeschbacher T. Cutting B. Allain F. H. Schubert M. Ernst B. (2013) Stabilization of branched oligosaccharides: Lewis(x) benefits from a nonconventional C-H–O hydrogen bond. J. Am. Chem. Soc. 135, 13464–13472 24001318 10.1021/ja4054702

[69] Kawashima H. Atarashi K. Hirose M. Hirose J. Yamada S. Sugahara K. Miyasaka M. (2002) Oversulfated chondroitin/dermatan sulfates containing GlcA β1/IdoA α1–3GalNAc(4,6-*O*-disulfate) interact with L- and P-selectin and chemokines. J. Biol. Chem. 277, 12921–12930 11821431 10.1074/jbc.M200396200

[70] Somers W. S. Tang J. Shaw G. D. Camphausen R. T. (2000) Insights into the molecular basis of leukocyte tethering and rolling revealed by structures of P- and E-selectin bound to SLe(X) and PSGL-1. Cell 103, 467–479 11081633 10.1016/s0092-8674(00)00138-0

[71] Pouyani T. Seed B. (1995) PSGL-1 recognition of P-selectin is controlled by a tyrosine sulfation consensus at the PSGL-1 amino terminus. Cell 83, 333–343 7585950 10.1016/0092-8674(95)90174-4

[72] Lever R. Page C. P. (2002) Novel drug development opportunities for heparin. Nat. Rev. Drug Discov. 1, 140–148 12120095 10.1038/nrd724

[73] Lever R. Hoult J. R. Page C. P. (2000) The effects of heparin and related molecules upon the adhesion of human polymorphonuclear leucocytes to vascular endothelium *in vitro*. Br. J. Pharmacol. 129, 533–540 10711352 10.1038/sj.bjp.0703099 PMC1571874

[74] Li J. Z. Lian E. C. (1988) Aggregation of human platelets by acidic mucopolysaccharide extracted from *Stichopus japonicus* Selenka. Thromb. Haemost. 59, 435–439 2973150

[75] Gray E. Mulloy B. Barrowcliffe T. W. (2008) Heparin and low-molecular-weight heparin. Thromb. Haemost. 99, 807–818 18449410 10.1160/TH08-01-0032

[76] Kaplan A. P. Joseph K. Silverberg M. (2002) Pathways for bradykinin formation and inflammatory disease. J. Allergy Clin. Immunol. 109, 195–209 11842287 10.1067/mai.2002.121316

[77] Blossom D. B. Kallen A. J. Patel P. R. Elward A. Robinson L. Gao G. Langer R. Perkins K. M. Jaeger J. L. Kurkjian K. M. Jones M. Schillie S. F. Shehab N. Ketterer D. Venkataraman G. Kishimoto T. K. Shriver Z. McMahon A. W. Austen K. F. Kozlowski S. Srinivasan A. Turabelidze G. Gould C. V. Arduino M. J. Sasisekharan R. (2008) Outbreak of adverse reactions associated with contaminated heparin. N. Engl. J. Med. 359, 2674–2684 19052120 10.1056/NEJMoa0806450 PMC3810025

[78] Li B. Suwan J. Martin J. G. Zhang F. Zhang Z. Hoppensteadt D. Clark M. Fareed J. Linhardt R. J. (2009) Oversulfated chondroitin sulfate interaction with heparin-binding proteins: new insights into adverse reactions from contaminated heparins. Biochem. Pharmacol. 78, 292–300 19389385 10.1016/j.bcp.2009.04.012 PMC2736791

[79] Kishimoto T. K. Viswanathan K. Ganguly T. Elankumaran S. Smith S. Pelzer K. Lansing J. C. Sriranganathan N. Zhao G. Galcheva-Gargova Z. Al-Hakim A. Bailey G. S. Fraser B. Roy S. Rogers-Cotrone T. Buhse L. Whary M. Fox J. Nasr M. Dal Pan G. J. Shriver Z. Langer R. S. Venkataraman G. Austen K. F. Woodcock J. Sasisekharan R. (2008) Contaminated heparin associated with adverse clinical events and activation of the contact system. N. Engl. J. Med. 358, 2457–2467 18434646 10.1056/NEJMoa0803200 PMC3778681

[80] Brown R. A. Lever R. Jones N. A. Page C. P. (2003) Effects of heparin and related molecules upon neutrophil aggregation and elastase release *in vitro*. Br. J. Pharmacol. 139, 845–853 12813008 10.1038/sj.bjp.0705291 PMC1573888

[81] Volpi N. (1997) Inhibition of human leukocyte elastase activity by chondroitin sulfates. Chem. Biol. Interact. 105, 157–167 9291994 10.1016/s0009-2797(97)00045-8

[82] Bartolucci C. Cellai L. Iannelli M. A. Lamba D. Liverani L. Mascellani G. Perola E. (1995) Inhibition of human leukocyte elastase by chemically and naturally oversulfated galactosaminoglycans. Carbohydr. Res. 276, 401–408 8542607 10.1016/0008-6215(95)00179-w

[83] van Die I. van Vliet S. J. Nyame A. K. Cummings R. D. Bank C. M. Appelmelk B. Geijtenbeek T. B. van Kooyk Y. (2003) The dendritic cell-specific C-type lectin DC-SIGN is a receptor for *Schistosoma mansoni* egg antigens and recognizes the glycan antigen Lewis x. Glycobiology 13, 471–478 12626400 10.1093/glycob/cwg052

[84] Miller K. E. Mukhopadhyay C. Cagas P. Bush C. A. (1992) Solution structure of the Lewis x oligosaccharide determined by NMR spectroscopy and molecular dynamics simulations. Biochemistry 31, 6703–6709 1353371 10.1021/bi00144a009

