# Supplementary material for: Fucosylated Chondroitin Sulfates from the Body Wall of the Sea Cucumber Holothuria forskali: CONFORMATION, SELECTIN BINDING, AND BIOLOGICAL ACTIVITY
Source: J Biol Chem. 2014 Aug 21;289(41):28284–98. doi: 10.1074/jbc.M114.572297 (PMC4192483; doi:10.1074/jbc.M114.572297)
Supplement: Supplemental Data [file supp_289_41_28284__index.html]

Fucosylated Chondroitin Sulfates from the Body Wall of the Sea Cucumber Holothuria forskali — Fucosylated Chondroitin Sulfates from H. forskali — Supplemental Data 

# Fucosylated Chondroitin Sulfates from the Body Wall of the Sea Cucumber *Holothuria forskali*

## Supplemental Data

**Files in this Data Supplement:**

- Supplemental Materials
