# Supplementary material for: Fucosylated Chondroitin Sulfates from the Body Wall of the Sea Cucumber Holothuria forskali: CONFORMATION, SELECTIN BINDING, AND BIOLOGICAL ACTIVITY
Source: J Biol Chem. 2014 Aug 21;289(41):28284–98. doi: 10.1074/jbc.M114.572297 (PMC4192483; doi:10.1074/jbc.M114.572297)
Supplement: Supplemental Data [file supp_M114.572297_Supplementary_material.pdf]

Fucosylated Chondroitin Sulfates from the Body Wall of the Sea Cucumber *Holothuria forskali*. Conformation, Selectin Binding and Biological Activity.

Charalambos Panagos, Derek S. Thomson, Claire Moss, Adam D. Hughes, Maeve S. Kelly, Yan Liu, Wengang Chai, Radhakrishnan Venkatasamy, Domenico Spina, Clive P. Page, John Hogwood, Robert J Woods, Barbara Mulloy, Charlie D. Bavington, Dušan Uhrín.

**Supplemental material**

Figure 1S: Dihedral angles during the course of MD simulation of the [→3)GalNAcβ4,6S(1→4)[Fucα2,4S (1→3)]GlcAβ(1→3)]$_4$ dodecasaccharide.

Figure 2S: Dihedral angles during the course of MD simulation of the [→3)GalNAcβ4,6S(1→4)[Fucα3,4S(1→3)]GlcAβ(1→3)]$_4$ dodecasaccharide.

Figure 3S: Dihedral angles of the sulfate groups during the course of MD simulation of fCS dodecasaccharides.

Table 1S. Dihedral angles of glycosidic linkages of fCS, CS and Le$^x$.

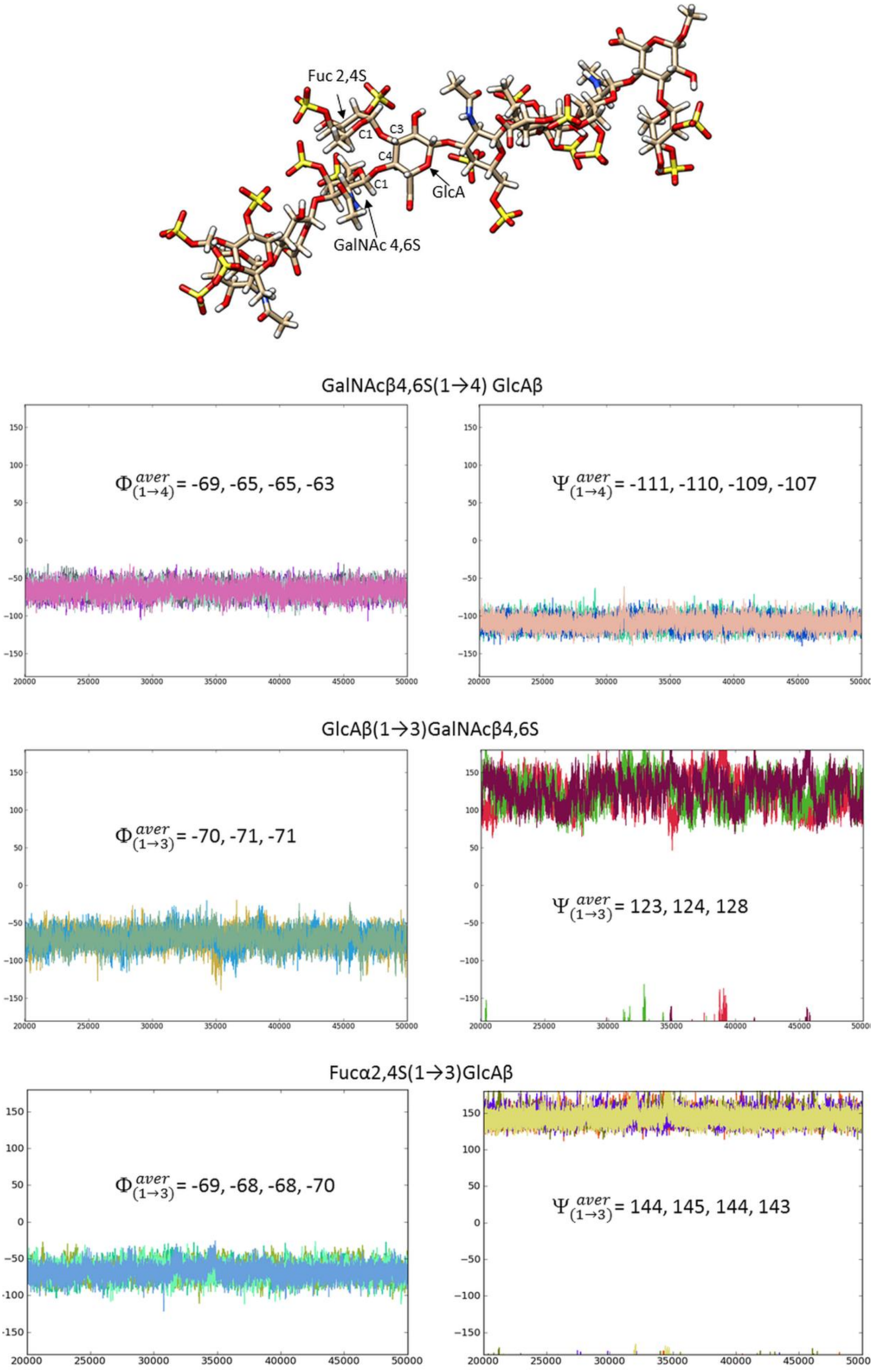


Figure 1S: Dihedral angles of the last 30,000 frames (30ns) of the MD simulation of the [→3)GalNAcβ4,6S(1→4)[Fucα2,4S(1→3)]GlcAβ(1→3)]$_4$ dodecasaccharide (closest to the average MD derive structure at the top) with GalNAc being the non-reducing residue. The average angles are given for the glycosidic bonds starting the non-reducing end. IUPAC definitions for the glycosidic $\Phi_{1\to X}$= $O_5$-$C_1$-O-$C'_X$ and $\Psi_{1\to X}$= $C_1$-O-$C_X$-$C'_{(X+1)}$ angles were used.

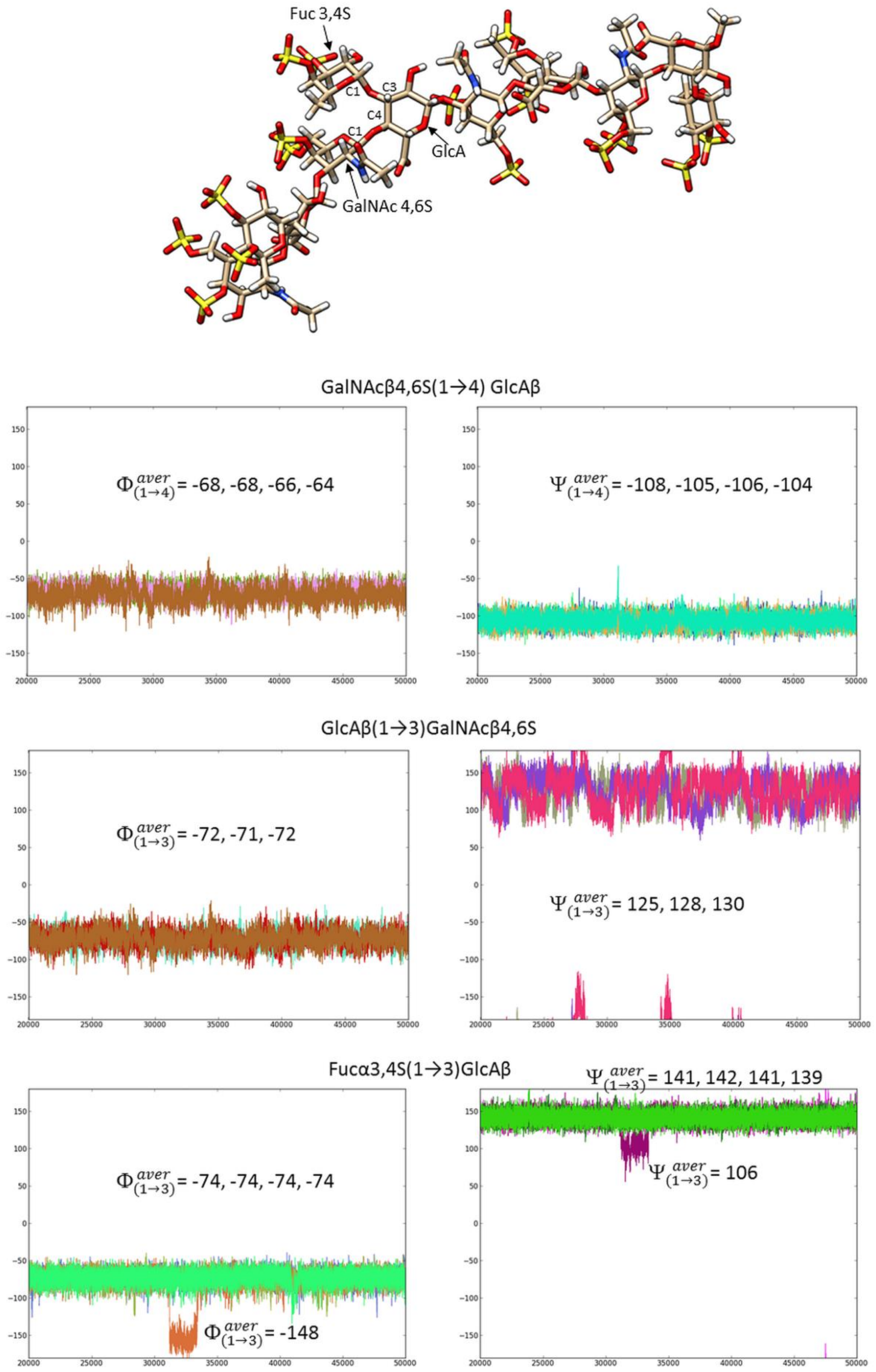


Figure 2S: Dihedral angles of the last 30,000 frames (30ns) of the MD simulation of the [→3)GalNAcβ4,6S(1→4) [Fucα3,4S(1→3)]GlcAβ(1→3)]$_4$ dodecasaccharide (closest to the average MD derive structure at the top) with GalNAc being the non-reducing residue. The average angles are given for the glycosidic bonds starting the non-reducing end. IUPAC definitions for the glycosidic $\Phi_{1\to X}$= $O_5$-$C_1$-O-$C'_X$ and $\Psi_{1\to X}$= $C_1$-O-$C_X$-$C'_{(X+1)}$ angles were used.

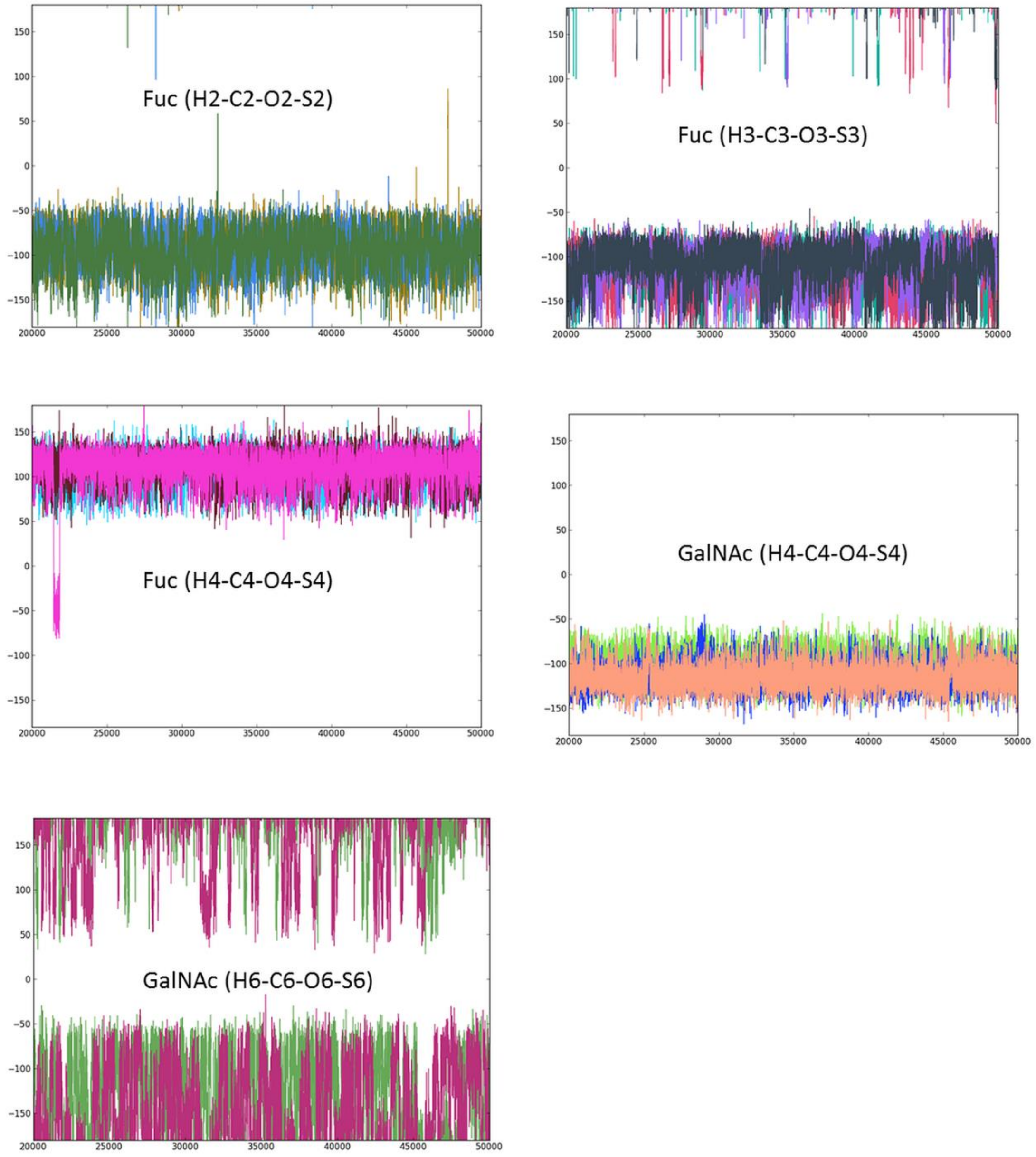


Figure 3S: Dihedral angles of the sulfate groups over the last 30,000 frames (30ns) of the MD simulation of the fCS dodecasaccharides. Only C6-sulfates show considerable rotational freedom.

Table 1S. Dihedral angles of glycosidic linkages of fCS, CS and Le$^x$

| Structure | dp[a] | Dihedral angle | β(1→4) | β(1→3) | α(1→3) |
|---|---|---|---|---|---|
| MD | 8 | Φ | **-66[b]** | **-71[c]** | **-69[d]** |
| | | Ψ | **-107[b]** | **126[c]** | **144[d]** |
| CS-ol | 5 | Φ | -67[e] | -61[f] | - |
| | | Ψ | -124[e] | 109[f] | - |
| de4S-CS | 6 | Φ | -73[g] | -72[h] | - |
| | | Ψ | -117[g] | 108[h] | - |
| X-ray CS fibre | 4 | Φ | -98 | -80 | - |
| | | Ψ | -174 | 107 | - |
| X-ray chondroitinase B CS complex | 4 | Φ | -69 | -86[i] | - |
| | | Ψ | -108 | 103[i] | - |
| [j]Le$^x$ | 2 | Φ | -66 | - | -73 |
| | | Ψ | -105 | - | 149 |
| [k]X-ray Le$^x$ | 2 | Φ | -76 | - | -75 |
| | | Ψ | -106 | - | 139 |

[a] degree of depolymerisation of the main chain

[b] this work, average over 4 linkages of fCS-**I** (Fuc 3,4) and fCS-**II** (Fuc 3,4) dodecasaccharide

[c] this work, average over 3 linkages of fCS-**I** (Fuc 3,4) and fCS-**II** (Fuc 3,4) dodecasaccharide

[d] this work, average over 4 linkages of fCS-**II** (Fuc 3,4) dodecasaccharide

[e] reduced pentasaccharide, average over 2 linkages (ref. 59)

[f] reduced pentasaccharide, the inner linkage linkages (ref. 59)

[g] average over 2 linkages (ref. 60)

[h] average over 3 linkages (ref. 60)

[I] average of 2 linkages (ref. 62)

[j] RDC based structure (ref. 58)

[k] average over two similar conformations (ref. 53)
